# Designing a Resilient Controller for Cancer Immunotherapy: Application to a Fractional‐Order Tumour‐Immune Model

**DOI:** 10.1049/syb2.70019

**Published:** 2025-06-05

**Authors:** Mohamadreza Homayounzade, Shayan Sajadian

**Affiliations:** ^1^ Mechanical Engineering Department Fasa University Fasa Iran

**Keywords:** biocontrol, cancer, control theory, Lyapunov methods, nonlinear control systems, robust control

## Abstract

In this paper, we propose a robust control method for the automatic treatment of targeted anti‐angiogenic molecular therapy based on multi‐input multi‐output (MIMO) nonlinear fractional and non‐fractional models using the backstepping (BS) approach. This protocol aims to eradicate tumour cells while preserving high levels of the body's natural effector cells and maintaining drug dosage within safe limits. The exponential stability of the controlled system is mathematically demonstrated using the Lyapunov theorem. Consequently, the tumour volume's convergence rate can be precisely controlled—a critical factor in cancer treatment. To fine‐tune the controller gains, a soft actor‐critic (SAC) algorithm within the framework of deep reinforcement learning (DRL) is employed, with a reward function designed based on the specific requirements of the system. Additionally, the Lyapunov theorem is used to mathematically verify the system's robustness against parametric uncertainty. Compared to state‐of‐the‐art approaches, the proposed scheme demonstrates superior long‐term performance, achieving complete tumour eradication and drug delivery convergence to zero within 50 days while preserving high effector cell levels.

## Graphical abstract

In this paper, we propose a robust control method for the automatic treatment of targeted anti‐angiogenic molecular therapy based on multi‐input multi‐output (MIMO) nonlinear fractional and non‐fractional models using the backstepping (BS) approach. By applying the Lyapunov theorem, the exponential stability of the controlled system has been mathematically demonstrated. Consequently, the tumour volume's convergence rate can be controlled—a crucial issue in cancer treatment. Moreover, the Lyapunov theorem is employed to mathematically verify the system's robustness against parametric uncertainty. Compared to state‐of‐the‐art approaches, the proposed scheme achieves superior long‐term performance.

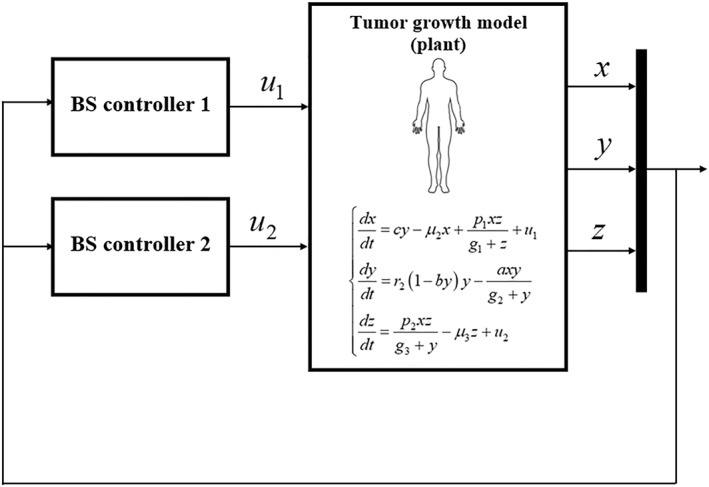

## 1 Introduction

Cancer is one of the most predominant causes of death, with 19.3 million new cases globally, and is responsible for 10 million deaths in 2020 [1]. There are several therapy approaches to treat cancer, the most effective of which include immunotherapy, chemotherapy, radiotherapy, and surgery.

The development of mathematical models to represent tumour‐immune dynamics has gained significant interest in recent years, as these models offer a cost‐effective and safe method for testing hypotheses, simulating complex system behaviours, and assessing therapy outcomes. The goal is to develop a suitable protocol for drug administration based on the model, although tumour growth is an intricate process involving complicated interactions.

The ordinary differential equation (ODE) can be explored for modelling the interactions among immune cells, cancer cells, and drug dosage regimens. A mathematical model for tumour‐immune dynamics is presented in ref. [2], where four differential equations represent tumour growth, immune response, and chemotherapy treatment. Additional mathematical models have been proposed in refs. [3, 4, 5, 6, 7, 8]. The Kirschner–Panetta model [9], known for its simplicity, is widely recognised for describing tumour‐immune interactions and has been used to test various control methods and develop drug administration scenarios.

Various control strategies have been proposed to effectively kill cancer cells while minimising the negative side effects of drugs on healthy cells. A number of widely used control techniques include optimal control theory [10, 11], multiple model adaptive control [12], neuro‐fuzzy control [13], sliding mode control [14], and state‐dependent Riccati equation control [15, 16]. A mathematical fractional‐order model for tumour‐immune dynamics is presented in ref. [17], where an optimal control approach is implemented. The controller minimises the tumour size and provides only uniformly boundedness of tumour volume. A multi‐objective optimisation approach is studied in ref. [18] to determine the optimal drug administration protocol for cancer patients. The effects of optimal control theory on reducing cancer cells are investigated in ref. [19]. Despite its stability, tumour cells cannot be completely eradicated and exhibit a 100‐day recurrent cycle. A cancer treatment model based on nonlinear fractional optimal control problems is proposed in ref. [20], where generalised shifted Legendre polynomials are used to approximate the solutions of the model. An open‐loop and a closed‐loop optimal controller are proposed in ref. [21], based on the calculus of variations and the state‐dependent Riccati equation, respectively, to design an optimal drug injection protocol.

However, in both refs. [20, 21], the stability and robustness of the controlled system have not been analysed.

When dealing with complex dynamic systems such as cancer, the free‐model control method serves as a valuable approach. Although classification and detection can be achieved through model‐free approaches such as machine learning (ML) and artificial intelligence (AI) techniques [22, 23, 24, 25], these methods have certain constraints. Although they can produce closed‐loop solutions based on historical information, they fall short in monitoring the real‐time changes of various physiological parameters during a patient's treatment and also heavily rely on the availability of data. In ref. [26], adaptive fuzzy control has been used to determine the optimal chemoimmunotherapy treatment scenario. Fuzzy functions, however, are designed based on training data, which can be inaccurate and is not readily available. In addition, fuzzy logic controllers are based on predefined rules and, as a result, lack robustness against topological changes in the system, only being able to respond within the scope of their initial rule base. A neural adaptive control approach is pursued in ref. [27], where Nussbaum functions and neural networks are employed to address the problem of unknown control direction and uncertain nonlinear dynamics, respectively. A combination of immunotherapy and chemotherapy is proposed in ref. [28], where the Takagi‐Sugeno fuzzy controller is derived based on the Stepanova nonlinear model. However, tumour cells cannot be entirely eliminated. In ref. [29], a novel closed‐loop fuzzy logic controller based on intuitionistic fuzzy sets and an invasive weed optimisation algorithm has been proposed. A novel bounded‐gain‐forgetting composite adaptation law is used in ref. [30] to address the parameter uncertainty problem and a nonlinear composite adaptive control strategy has been implemented.

In ref. [31], using a recurrent neural network, a mathematical model of the cancer dynamics has been identified, and then the sliding mode control approach was employed to design the controller. However, the controller suffers from chattering. Optimal sliding mode control (SMC) is applied in ref. [32] to optimise drug delivery in chemotherapy, where the effects of obesity are also considered. Two non‐linear controllers using feedback linearisation and sliding mode approaches are proposed in ref. [33]. In addition, a genetic algorithm and a fuzzy tuner were employed to optimise the therapy parameters. The controller, however, lacks robustness against uncertainties and achieves only basic stability, which is considered the weakest form of stability.

Another powerful control approach widely used by researchers is the backstepping control method, which provides a systematic and flexible procedure for designing controllers for nonlinear system models. In ref. [34], for the multiple‐input multiple‐output (MIMO) cancer immunotherapy system, an adaptive fuzzy backstepping controller was proposed. In ref. [35], a model‐free controller, using an adaptive backstepping control approach and Legendre polynomials, was designed for cancer treatment; in contrast, Laguerre polynomials were utilised to deal with the uncertainty estimations in ref. [36].

In ref. [37], a mixed chemotherapy‐immunotherapy treatment protocol was developed by implementing a pseudo‐spectral control method. In ref. [38], a backstepping controller is combined with a sliding mode approach to develop the cancer treatment regimen.

A systematic review of 105 studies on optic pathway glioma management, presented in ref. [39], analysed patient demographics, treatment types, outcomes, and complications, highlighting significant limitations related to sample size, study heterogeneity, and challenges in assessing paediatric visual outcomes. An optimised approach utilising generalised Lerch polynomials is presented in ref. [40] to solve the fractional model of CAR‐T cell therapy for T‐cell leukaemia. This method effectively predicts a decrease in leukaemic T‐cells and suggests potential for enhanced treatment through sequential CAR‐T cell doses. In ref. [41], the development of a positive input dynamics method for tumour growth control was presented.

Optimal control theory is applied in ref. [42] to develop personalised cancer treatment plans, comparing strategies, such as Interior Point OPTimizer, State‐Dependent Riccati Equation, and Active Set Range Estimation to optimise chemotherapy and immunotherapy integration, aiming to minimise side effects and maximise therapeutic efficacy while considering individual patient differences. The mathematical modelling of cancer treatment is explored in ref. [43], focusing on chemotherapy as an optimal control problem to maximise tumour cell death while preserving normal cells, incorporating pharmacokinetics and validating results numerically to address drug resistance and toxicity challenges. Tumour‐immune cell growth is investigated in ref. [44] under chemotherapy drug diffusion using a Caputo fractional‐order model, employing the modified homotopy perturbation method to analyse spatiotemporal dynamics and highlighting the significant role of fractional order in understanding tumour‐immune interactions. In ref. [45] a novel control system combining a delayed‐output state observer and a backstepping‐based feedback controller is designed to address instability from measurement delays and positive input constraints in tumour growth control.

In light of the above‐mentioned problems, in this paper we develop a robust control method for the automatic treatment of targeted anti‐angiogenic molecular therapy based on multi‐input multi‐output (MIMO) nonlinear fractional and non‐fractional models using the backstepping (BS) approach. The BS approach is a recursive design procedure for stabilising the origin of a system in strict‐feedback form and guarantees the global asymptotic stability of feedback systems. The exponential stability of the controlled system is proved mathematically using the Lyapunov theorem. As a result, the convergence rate of the tumour volume can be controlled, which is a very important issue in cancer treatment. Moreover, the robustness of the system against parametric uncertainties is verified mathematically using the Lyapunov theorem. The main novelties of the proposed method are as follows:

1. Unlike refs. [22, 23, 24, 25, 26, 27, 28, 29, 31, 34], which rely on data‐intensive machine learning models and rule‐based fuzzy approaches—both of which may face challenges in immunotherapy applications due to limited data availability and lack of robustness to changes and uncertainties—our approach leverages a backstepping (BS) method. This systematic, recursive design process ensures stability and robustness without the dependency on large datasets. In addition, unlike these model‐free approaches, the backstepping method is based on mathematical models, providing more physiological details that help physicians make more informed decisions.
2. Unlike refs. [10, 11, 12, 13, 14, 15, 16, 17, 18, 19, 20, 21, 22, 23, 24, 25, 26, 27, 28, 29, 30, 31, 32, 33, 34, 35, 36, 37, 38], where stability analysis is either overlooked or the proposed controller is asymptotically stable, our stability analysis is conducted mathematically using the Lyapunov theorem, proving that the controlled system is exponentially stable. This exponential stability enables more predictable convergence, allowing the time required for tumour cell eradication to be precisely determined and controlled. Consequently, clinicians can plan treatment timelines more effectively, ensuring a more targeted and reliable approach to cancer therapy.
3. Unlike refs. [10, 11, 12, 13, 14, 15, 16, 18, 19, 20, 21, 30, 31, 32, 33, 34, 35, 36, 37], which only considers the integer model of cancer dynamics, our approach applies both integer and fractional order models. Given that cancer dynamics exhibit hereditary properties and may have a lower diffusion rate, fractional order models can more effectively represent these dynamics. Additionally, the stability of the fractional order model has been mathematically proven using the Lyapunov theorem.
4. The system's robustness is either analysed solely through simulations or disregarded completely in refs. [18, 19, 20, 21, 26, 27, 28, 29, 35, 37]. In this paper, we conduct a rigorous mathematical analysis of the system's robustness using the Lyapunov theorem, demonstrating that the controlled system is bounded input‐bounded output (BIBO) stable when subjected to perturbations. Additionally, the robustness of the proposed controller is investigated through simulations, which examine 10%–100% uncertainties in the system parameters. The simulation results confirm that the closed‐loop system remains robust against parametric uncertainties.
5. In this article, the proposed immunotherapy protocol completely eradicates the tumour cell concentration in approximately 50 days, whereas the protocols suggested by refs. [34, 38] require roughly 300 days to achieve similar results. In addition, unlike ref. [35], where it takes 150 days to eliminate the cancer cells and the controller fails to converge to zero for drug delivery—resulting in a permanent drug injection rate of 650 cells/mL—our controller demonstrates superior long‐term performance, with both tumour cells and drug delivery converging to zero within 50 days.
6. Leveraging the training capabilities of deep neural networks (DNN), a DRL‐based tuning mechanism is implemented to optimise the proposed backstepping controller.

The rest of the paper is organised as follows: in Section 2, the tumour growth model is presented. In Section 3, the controller is presented. In Section 4, the stability of the equilibrium point and the BIBO (bounded input‐bounded output) stability of the system exposed to external disturbances and uncertainties are analysed mathematically using the Lyapunov theorem. In Section 5, the control method is applied to the fractional order model, and in Section 6, simulation results are presented. Finally, this paper is concluded in Section 7.

## 2 Tumour Growth Model

The tumour immune‐tumour model is presented in subsection 2.1, and the error systems are presented in subsection 2.2.

### 2.1 System Modelling

The differential equations governing the tumour immune‐tumour model are derived from the following [9, 35]:

(1a) $$\frac{dx}{dt}=cy-\mu_{2}x+\frac{p_{1}xz}{g_{1}+z}+u_{1},$$

(1b) $$\frac{dy}{dt}=r_{2}\left(1-by\right)y-\frac{axy}{g_{2}+y},$$

(1c) $$\frac{dz}{dt}=\frac{p_{2}xz}{g_{3}+y}-\mu_{3}z+u_{2},$$

in which c is the antigenicity of the tumour, $\mu_{2}$ is the immune cells' death rate, $g_{1}$ is the half‐sat for proliferation term, $g_{2}$ is the half‐sat for cancer clearance, b is the logistic growth of cancer capacity, $\mu_{3}$ is the effector molecule's half‐life, $r_{2}$ is the growth rate of cancer, $p_{1}$ is the immune cells' proliferation rate, $p_{2}$ is the effector molecules' production rate, a is the cancer clearance term, and $g_{3}$ is the half‐sat of production. Moreover, the model illustrates the mutual interactions among three key elements: the concentration of activated immune cells (x), often referred to as effector cells—such as cytotoxic T‐cells, macrophages, and natural killer cells that are toxic to tumour cells—the concentration of tumour cells (y), and the level of Interleukin‐2 (z). In addition, $u_{1}$ is a treatment term which represents an external source of effector cells such as lymphokine‐activated killer (LAK), and $u_{2}$ shows the influence of tumour infiltrating lymphocyte cells [9, 35].

### 2.2 Error Systems

In this subsection, first, the design procedure of the controller using the BS technique is described, and then the proposed controller is presented in Theorem 1.

Let us define the variable e by the following:

(2) $$e=\alpha x-x^{*},$$

where $x^{*}$ represents the desired magnitude of x which will be designed properly in Section 3 through the BS approach. Considering the definition of e by Equation (2), we can rewrite Equation (1b) as follows:

(3) $$\dot{y}=r_{2}\left(1-by\right)y-\frac{1}{\alpha }\frac{ay}{g_{2}+y}\left(e+x^{*}\right).$$

As a result, $x^{*}$ can act as a guiding variable for controlling state y. In Section 3, the desired magnitude $x^{*}$ is designed properly using the BS approach such that the state y asymptotically converges to zero if x tends to $x^{*}$.

Differentiating definition (2), we obtain the following:

(4) $$\dot{e}=\alpha \dot{x}-{\dot{x}}^{*}.$$

Substituting Equation (1a) in the result, we obtain the following:

(5) $$\dot{e}=\alpha \left(cy-\mu_{2}x+\frac{p_{1}xz}{g_{1}+z}+u_{1}\right)-{\dot{x}}^{*}.$$

As a result, the control input should be designed in a way that the state e converges to zero, that is, x tends to $x^{*}$.

Remark 1 The controller method using the BS technique is as follows:

1. The desired variable $x^{*}$ is appropriately designed such that the state y exponentially converges to zero if x tends to $x^{*}$.
2. The input control law $u_{1}$ is appropriately designed so that the tracking error e exponentially converges to zero, that is, x tends to $x^{*}$.
3. The input control law $u_{2}$ is appropriately designed so that the state z exponentially converges to zero.

## 3 Controller Design

The BS technique is used to design the desired magnitude $x^{*}$ as follows:

(6) $$x^{*}=\frac{\alpha }{a}\left(g_{2}+y\right)\left(r_{2}\left(1-by\right)+k_{1}\right),$$

where the constant $k_{1}$ is the positive control gain. Clearly Equation (6) can be rearranged as follows:

(7) $$x^{*}=Ay^{2}+By+D,$$

where

(8) $$A=-\frac{\alpha r_{2}b}{a},B=\frac{\alpha r_{2}}{a}-\frac{\alpha r_{2}bg_{2}}{a}+\frac{\alpha k_{1}}{a},\;D=\frac{r_{2}\alpha }{a}g_{2}+\frac{k_{1}\alpha }{a}g_{2}.$$

As mentioned in Remark 1, the costate $x^{*}$ is designed so that the state y exponentially converges to zero when x approaches $x^{*}$.

Substituting $x^{*}$ by Equation (6) in Equation (3), we obtain the following equation:

(9) $$\dot{y}=-\frac{1}{\alpha }\frac{ay}{g_{2}+y}e-\frac{1}{\alpha }k_{1}y.$$

Differentiating Equation (7) with respect to time, we obtain the following equation:

(10) $${\dot{x}}^{*}=\left(2Ay+B\right)\;\dot{y}.$$

Substituting Equation (9) in Equation (10), we obtain the following equation:

(11) $${\dot{x}}^{*}=\left(2Ay+B\right)\;\left(-\frac{1}{\alpha }\frac{ay}{g_{2}+y}e-\frac{1}{\alpha }k_{1}y\right).$$

Substituting Equations (1a) and Equation (11) in Equation (4), we obtain the following equation:

(12) $$\dot{e}=\alpha cy-\alpha \mu_{2}x+\frac{\alpha p_{1}xz}{g_{1}+z}+\alpha u_{1}-\left(2Ay+B\right)\;\left(-\frac{1}{\alpha }\frac{ay}{g_{2}+y}e-\frac{1}{\alpha }k_{1}y\right).$$

Let us design the control input $u_{1}$ by the following equation:

(13) $$u_{1}=-cy+\mu_{2}x+\frac{1}{\alpha }\left(2Ay+B\right)\;\left(-\frac{1}{\alpha }\frac{ay}{g_{2}+y}e-\frac{1}{\alpha }k_{1}y\right)-\frac{p_{1}xz}{g_{1}+z}+\frac{ay}{g_{2}+y}y-k_{2}e.$$

where A,B are previously defined by Equation (8) and $k_{1},k_{2}$ represent control gains.

The control input law $u_{1}$ is designed using the feedback linearisation technique to ensure that the tracking error e exponentially converges to zero. A detailed explanation of the feedback linearisation technique is provided in Remark 2.

Substituting Equation (13) in Equation (12), we obtain the following equation:

(14) $$\dot{e}=\alpha \frac{ay^{2}}{g_{2}+y}-\alpha k_{2}e.$$

Let us design the control input $u_{2}$ by the following equation:

(15) $$u_{2}=-\frac{p_{2}xz}{g_{3}+y}+\mu_{3}z-k_{3}z.$$

where $k_{3}$ represents the control gain.

The input control law $u_{2}$ is designed using the feedback linearisation technique to ensure that the state z exponentially converges to zero. A detailed explanation of the feedback linearisation technique is provided in Remark 2.

Substituting Equation (15) in Equation (1c), we obtain the following equation:

(16) $$\dot{z}=-k_{3}z.$$

Remark 2 Feedback linearisation is a nonlinear control technique that transforms a nonlinear system into an equivalent linear system by applying a nonlinear state and input transformation. The core idea is to algebraically cancel the system's nonlinearities, enabling the use of conventional linear control methods. This is achieved by differentiating the system output until the control input explicitly appears, allowing for the design of a linearising control law. By doing so, the closed‐loop system behaves like a fully or partially linear system, simplifying stability and tracking control. While highly effective for precise control applications, feedback linearisation relies on an accurate system model and can be sensitive to uncertainties and external disturbances.

**FIGURE 1:**
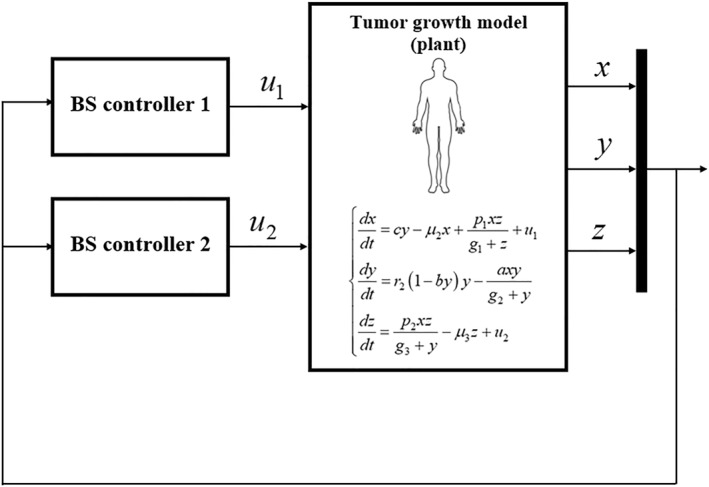
The schematic of the controlled system.

The schematic of the closed‐loop system is presented in Figure 1.

Theorem 1 *The control inputs derived from* Equations (13) and (15) *exponentially stabilise the system, ensuring that the states* y *and* z *, as well as the costate* e *, exponentially converge to zero,* that is, $\left|\;y\left(t\right)\right|\leq \beta e^{-\frac{1}{2}\;\gamma t},\left|\;z\left(t\right)\right|\leq \beta \;e^{-\frac{1}{2}\;\gamma t},$ $\left|e\left(t\right)\right|\leq \beta e^{-\frac{1}{2}\;\gamma t},$ *where the positive constant* γ *adjusts the rate of convergence of system errors.*

Remark 3 It will be shown that by increasing the control gain $k_{1},k_{2},k_{3}$, and/or decreasing the magnitude of α the rate of convergence of tumour volumes and inhibitor level decreases.

## 4 Stability Analysis

In subsection 4.1, we analyse the stability of the equilibrium point. Then, the BIBO stability of the controlled system in the presence of parametric uncertainties is analysed through the Lyapunov theorem. Consequently, the robustness of the system is assured using a high gain controller. The proposed BS controller is summarised in Table 1.

**TABLE 1:** BS controller design.

Tumour growth model:	$\left\{\begin{array}{l} \dot{x}=cy-\mu_{2}x+\frac{p_{1}xz}{g_{1}+z}+u_{1}\\ \dot{y}=r_{2}\left(1-by\right)y-\frac{axy}{g_{2}+y}\\ \dot{z}=\frac{p_{2}xz}{g_{3}+y}-\mu_{3}z+u_{2} \end{array}\right.$ x: concentration of effector cells y: concentration of tumour cells z: concentration of single cancer site
Input controller:	$u_{1}=-cy+\mu_{2}x+\left(2Ay+B\right)\;\left(-\frac{ay}{g_{2}+y}e-k_{1}y\right)-\frac{p_{1}xz}{g_{1}+z}+\frac{ay}{g_{2}+y}y-k_{2}e$ $u_{2}=-\frac{p_{2}xz}{g_{3}+y}+\mu_{3}z-k_{3}z$ $k_{1},k_{2},k_{3}:$ control gains
Stability:	‐ Exponentially stable, ‐ BIBO stable.

Using a similar approach as in Section 3, we can write the system error dynamics in the presence of uncertainties in system parameters and time varying disturbance as follows:

(17a) $$\dot{y}=-\frac{ay}{g_{2}+y}e-k_{1}y+d_{1},$$

(17b) $$\dot{e}=\frac{ay^{2}}{g_{2}+y}-k_{2}e+d_{2},$$

(17c) $$\dot{z}=-k_{3}z+d_{3},$$

where $d_{1},d_{2},d_{3}$ represent the deviation of system parameters from their nominal values.

Considering definition (26) and Equations ((17a), (17b), (17c)), in the presence of uncertainties, the closed‐loop error systems can be rearranged as follows:

(18) $$\dot{X}=f\left(X\right)+u_{d}\left(t,X\right)$$

where

(19a) $$X=\left[\begin{array}{l} y\\ e\\ z \end{array}\right],$$

(19b) $$f\left(X\right)=\left[\begin{array}{c} -\frac{ay}{g_{2}+y}e-k_{1}y\\ \frac{ay^{2}}{g_{2}+y}-k_{2}e\\ -k_{3}z \end{array}\right],u_{d}\left(t,X\right)=\left[\begin{array}{l} d_{1}\\ d_{2}\\ d_{3} \end{array}\right]$$

The perturbation term $u_{d}\left(t,X\right)$ could result from modelling errors, ageing, or uncertainties and disturbances, which exists in any realistic problem. In a typical situation, we do not know $u_{d}\left(t,X\right)$, but we know some information about it, like knowing an upper bound on $\left\|u_{d}\left(t,X\right)\right\|$. Here, we represent the perturbation as an additive term on the right‐hand side of the state equation. Uncertainties that do not change the system's order can always be represented in this form. For if the perturbated right‐hand side is some function $\overset{∼}{f}\left(X\right)$, then by adding and subtracting f(X), we can rewrite the right‐hand side as $\overset{∼}{f}\left(X\right)=f\left(X\right)+\left[\overset{∼}{f}\left(X\right)-f\left(X\right)\right]$ and define $u_{d}\left(t,X\right)=\overset{∼}{f}\left(X\right)-f\left(X\right)$.

In Section 4.1, it is shown that the nominal system (18) has a uniformly exponentially stable equilibrium point at the origin. In Section 4.2, it is shown that in the case $u_{d}\left(t,0\right)\neq 0$, the origin will not be an equilibrium point of the perturbed system. In this case, we study BIBO stability of the system.

### 4.1 Exponential Stability of Nominal System

If $u_{d}\left(t,0\right)=0$ (in the presence of certain parameters) the perturbed system (18) has an equilibrium point at the origin. The main results regarding this research are mentioned in the form of the following theorem. In this case, we analyse the stability behaviour of the origin as an equilibrium point of the perturbed system.

Remark 4 The Lyapunov theorem provides a robust framework for analysing the stability of an equilibrium point in dynamic systems. It is particularly advantageous for nonlinear systems, as it does not require linearisation or small‐signal approximations. By constructing a Lyapunov function—an energy‐like measure—it enables the assessment of both local and global stability. The theorem provides explicit and systematic conditions for stability, offering clarity in analysis. Moreover, it accounts for uncertainties and disturbances, ensuring robust stability. Overall, the Lyapunov theorem is a powerful tool for evaluating and ensuring the stability of equilibrium points in diverse and complex systems.

The control inputs By Equations (13) and (15), exponentially stabilise the equilibrium point of the nominal system, that is

(20) $$\lim_{t\to \infty }y=0,\lim_{t\to \infty }z=0,\lim_{t\to \infty }e=0.$$

Select the Lyapunov function

(21) $$V=\frac{1}{2\alpha }e^{2}+\frac{1}{2}y^{2}+\frac{1}{2}z^{2}.$$

Differentiating Equation (21) with respect to time to obtain

(22) $$\dot{V}=\frac{1}{\alpha }e\;\dot{e}+y\;\dot{y}+z\;\dot{z}.$$

Substituting Equations (9), (14), (16) in Equation (22) to obtain

(23)

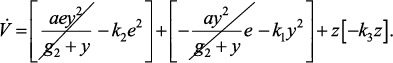




Simplifying equal terms in Equation (23) we obtain the following equation:

(24) $$\dot{V}=-k_{1}y^{2}-k_{2}e^{2}-k_{3}z^{2}.$$

Equation (24) ensures $\dot{V}\leq 0$. Considering that

(25) $$V=\frac{1}{2}X^{T}M\;X.$$

where

(26) $$M=\left[\begin{array}{ccc} \frac{1}{\alpha } & 0 & 0\\ 0 & 1 & 0\\ 0 & 0 & 1 \end{array}\right]$$

**Definition.** Let us define the Euclidean norm for an arbitrary vector X as

(27) $${\left\|X\right\|}^{2}=X^{T}X.$$

Considering that V is a positive definite function we have

(28) $$\lambda_{1}{\left\|X\right\|}^{2}\leq V\leq \lambda_{2}{\left\|X\right\|}^{2},$$

where $\lambda_{1},\lambda_{2}$ represent respectively the minimum and maximum eigenvalues of matrix M.

Considering Equation (24) we have the following equation:

(29) $$\dot{V}\leq -\frac{\gamma }{\lambda_{1}}V,$$

where $\gamma =\min\left\{k_{1},k_{2},k_{3}\right\}$ is a positive constant.

Integrating both sides of Equation (29) to find

(30) $$\int_{V\left(0\right)}^{V\left(t\right)}\frac{dV}{V}\leq -\frac{\gamma }{\lambda_{1}}\int_{0}^{t}dt\;\Rightarrow \mathrm{Ln}\left(\frac{V\left(t\right)}{V\left(0\right)}\right)\leq -\frac{\gamma }{\lambda_{1}}t,$$

as well

(31) $$V\left(t\right)\leq V\left(0\right)\;e^{-\frac{\gamma }{\lambda_{1}}t}.$$

Considering Equation (28), Equation (29) can be considered as follows:

(32) $$\left\|X\left(t\right)\right\|\leq \frac{\lambda_{2}}{\lambda_{1}}\left\|X\left(0\right)\right\|\;e^{-\frac{1}{2}\;\gamma t}.$$

Considering definitions (19a) and Equation (32) results

(33) $$\left|y\left(t\right)\right|\leq \beta \;e^{-\frac{1}{2}\;\gamma t},\left|z\left(t\right)\right|\leq \beta \;e^{-\frac{1}{2}\;\gamma t},\left|e\left(t\right)\right|\leq \beta \;e^{-\frac{1}{2}\;\gamma t},$$

where β is a positive constant. Equation (33) implies that states y(t),e(t) and z(t) exponentially converge to zero, that is $\lim_{t\to \infty }\;y\left(t\right)=0,\lim_{t\to \infty }\;z\left(t\right)=0,\lim_{t\to \infty }\;e\left(t\right)=0$

Remark 5 Considering Equation (33) it can be observed that the parameter γ specifies the rate of convergence of y(t),z(t). Consequently, by adjusting the control gains $k_{1},k_{2},k_{3}$, we can adjust the parameter γ and as a consequence, we can control the rate of convergence of states y and z.

Remark 6 Considering $x^{*}$ from Equation (7) and the fact that $\lim_{t\to \infty }\;y\left(t\right)=0$, it follows that $x^{*}\left(t\right)$ tends to D as time increases. Specifically, $x^{*}\left(t\right)$ approaches $\alpha \left(\frac{r_{2}}{a}g_{2}+\frac{k_{1}}{a}g_{2}\right)$ over time. The parameters $r_{2},a,g_{2}$ are inherent system parameters and cannot be directly designed. However, by selecting an appropriate value for the constant α, the steady‐state magnitude of the state x and the costate $x^{*}\left(t\right)$ can be regulated. By examining the definition of $u_{1}$ and $u_{2}$ in Equations (13) and (15) and noting $\lim_{t\to \infty }\;y\left(t\right)=0$, $\lim_{t\to \infty }\;e\left(t\right)=0$ and $\lim_{t\to \infty }\;z\left(t\right)=0$, it can be concluded that the steady‐state magnitude of the control input $u_{2}$ converges to zero. Furthermore, the magnitude of $u_{1}$ can be reduced by increasing α and decreasing the control parameter $k_{1}$. However, as mentioned in Remark 2, increasing α and decreasing $k_{1}$ will also result in a slower rate of convergence. Therefore, a trade off must be made between the rate of convergence and the allowable magnitude of the input $u_{1}$. In Section 5.1, we employ an optimsation technique to determine the optimal control gains.

### 4.2 Robustness of the System Against Parametric Uncertainties and Time‐Varying Disturbance

Let us turn now to the more general case when we do not know that $u_{d}\left(t,0\right)\neq 0$. The origin X=0 may not be an equilibrium point of the perturbed system (18), nor should we expect the solution of the perturbed system to approach the origin as t→∞. The main results regarding this research are mentioned in the form of the following theorem. This subsection examines how the closed‐loop system behaves when the system parameters are uncertain. The nominal value of system parameters is denoted by the “
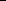
” notation.

Theorem 2 *Let* X=0 *be an exponentially stable equilibrium point of the nominal system* $\dot{X}=f\left(X\right)$ *. Let* V(X) *be a Lyapunov function of the nominal system that satisfies* (36) *in* D *, where* $D=\left\{X\in R^{3}|\;\left\|X\right\|<r\right\}$ *. Suppose the perturbation term* $u_{d}\left(t,X\right)$ *satisfies*

(34) $$\left\|u_{d}\left(t,X\right)\right\|\leq \delta <\frac{\lambda_{3}}{\lambda_{4}}\sqrt{\frac{\lambda_{1}}{\lambda_{2}}}\theta \;r$$

For the tumour growth system modelled by Equations ((1a), (1b), (1c)), the controllers are given by the following equation:

(35a) $$u_{1}=-\bar{c}y+{\bar{\mu }}_{2}x+\left(2\bar{A}y+\bar{B}\right)\;\left(-\frac{\bar{a}y}{g_{2}+y}e-k_{1}y\right)-\frac{{\bar{p}}_{1}xz}{g_{1}+z}+\frac{\bar{a}y}{g_{2}+y}y-k_{2}e,$$

(35b) $$u_{2}=-\frac{{\bar{p}}_{2}xz}{g_{3}+y}+{\bar{\mu }}_{3}z-k_{3}z,$$

guarantees that the system is BIBO stable in the presence of uncertainties and time varying disturbance.

We use V(x) calculated by Equation (21) as a Lyapunov function for the perturbed system (18) which satisfies

(36) $$\begin{array}{l} \lambda_{1}{\left\|X\right\|}^{2}\leq V\left(X\right)\leq \lambda_{2}{\left\|X\right\|}^{2}\\ \frac{\partial V}{\partial X}f\left(X\right)\leq -\lambda_{3}{\left\|X\right\|}^{2}\\ \left\|\frac{\partial V}{\partial X}\right\|\leq \lambda_{4}\left\|X\right\| \end{array}$$

For all X∈D for some positive constants $\lambda_{1},\lambda_{2},\lambda_{3}$ and $\lambda_{4}$.

The derivative of V(X) along the trajectories of (18) satisfies

(37) $$\begin{array}{l} \dot{V}\left(X\right)\leq -\lambda_{3}{\left\|X\right\|}^{2}+\left\|\frac{\partial V}{\partial X}\right\|\;\left\|g\left(t,X\right)\right\|\\ \leq -\lambda_{3}{\left\|X\right\|}^{2}+\lambda_{4}\delta \left\|X\right\|\\ =-\left(1-\theta \right)\lambda_{3}{\left\|X\right\|}^{2}-\theta \lambda_{3}{\left\|X\right\|}^{2}+\lambda_{4}\delta \left\|X\right\|\\ \leq -\left(1-\theta \right)\lambda_{3}{\left\|X\right\|}^{2},\forall \;\left\|X\right\|\geq \delta \lambda_{4}/\theta \lambda_{3} \end{array}$$

Choosing θ<1, we have

(38) $$\dot{V}\leq -\lambda_{3}{\left\|X\right\|}^{2},\forall \;\left\|X\right\|\geq \mu >0$$

where $\mu =\delta \lambda_{4}/\theta \lambda_{3}$.

It is evident from the first of Equation (37) that if $V\geq \lambda_{2}\mu^{2}$, then ‖X‖≥μ, and

(39) $$\dot{V}\leq -\frac{\lambda_{3}}{\lambda_{2}}V\;\Rightarrow \;V\left(X\left(t\right)\right)\leq e^{-\left(\lambda_{3}/\lambda_{2}\right)\left(t-t_{0}\right)}\;V\left(X\left(t_{0}\right)\right)$$

Hence

(40) $$\begin{array}{cc} & \left\|X\left(t\right)\right\|\leq {\left(\frac{V\left(X\left(t\right)\right)}{\lambda_{1}}\right)}^{1/2}\leq {\left(\frac{1}{\lambda_{1}}e^{-\left(\lambda_{3}/\lambda_{2}\right)\left(t-t_{0}\right)}V\left(X\left(t_{0}\right)\right)\right)}^{1/2}\\ & \;\leq {\left(\frac{1}{\lambda_{1}}e^{-\left(\lambda_{3}/\lambda_{2}\right)\left(t-t_{0}\right)}\lambda_{2}{\left\|X\left(t_{0}\right)\right\|}^{2}\right)}^{1/2}\\ & \;={\left(\frac{\lambda_{2}}{\lambda_{1}}\right)}^{1/2}e^{-\left(\lambda_{3}/2\lambda_{2}\right)\left(t-t_{0}\right)}\left\|X\left(t_{0}\right)\right\|=\lambda e^{-\gamma \left(t-t_{0}\right)}\left\|X\left(t_{0}\right)\right\| \end{array}$$

The above inequality remains valid over the interval $\left[t_{0},t_{0}+T\right]$, provided $V\geq \lambda_{2}\mu^{2}$. Additionally, for $t\geq t_{0}+t$, we obtain

(41) $$\left\|X\left(t\right)\right\|\leq {\left(\frac{V\left(X\left(t\right)\right)}{\lambda_{1}}\right)}^{1/2}\leq {\left(\frac{\lambda_{2}\mu^{2}}{\lambda_{1}}\right)}^{1/2}=\lambda \mu$$

For t≥0, any X∈D, and a positive constant θ<1, it follows that for all $\left\|X\left(t_{0}\right)\right\|<\sqrt{\lambda_{1}/\lambda_{2}}r$, the solution X(t) of the perturbed system (18) fulfils the condition

(42) $$\left\|X\left(t\right)\right\|\leq \lambda \;\exp\left[-\gamma \left(t-t_{0}\right)\right]\;\left\|X\left(t_{0}\right)\right\|,\forall \;t_{0}\leq t<t_{0}+T$$

and

(43) $$\left\|X\left(t\right)\right\|\leq b,\forall \;t\geq t_{0}+T$$

Remark 7 The ultimate magnitude of ‖X(t)‖, that is b, can be decreased by increasing the magnitude of control gains.

For a certain finite T, such that

(44) $$\lambda =\sqrt{\frac{\lambda_{2}}{\lambda_{1}}},\gamma =\frac{\left(1-\theta \right)\lambda_{3}}{2\lambda_{2}},b=\frac{\lambda_{4}}{\lambda_{3}}\sqrt{\frac{\lambda_{2}}{\lambda_{1}}}\frac{\delta }{\theta }$$

Consider $W=\sqrt{V\left(X\right)}$, when V(x)≠0, use W˙=V˙2V and Equation (37) and consider Equation (36) to obtain the following equation:

(45) $$\begin{array}{cc} & \dot{W}\leq \frac{-\lambda_{3}\;{\left\|X\left(t\right)\right\|}^{2}+\left\|X\left(t\right)\right\|\;\delta }{\;\sqrt{\lambda_{1}}\;\left\|X\left(t\right)\right\|}\\ & \;\leq -\frac{1}{\sqrt{\lambda_{1}}}\lambda_{3}\;\left\|X\left(t\right)\right\|+\frac{1}{\sqrt{\lambda_{1}}}\delta \\ & \leq -\frac{\lambda_{3}}{\lambda_{2}\sqrt{\lambda_{1}}}\;W+\frac{1}{\sqrt{\lambda_{1}}}\delta . \end{array}$$

When V(X,t)=0, the right derivative of W is calculated by using the following equation:

(46) $$\begin{array}{ll} D^{+}W & =\lim\;\underset{h\to 0^{+}}{\sup}\;\frac{1}{h}\left[W\left(t+h,X\left(t+h\right)\right)-W\left(t,X\left(t\right)\right)\right]\\ & =\lim\;\underset{h\to 0^{+}}{\sup}\;\frac{1}{h}\sqrt{V\left(t+h,X\left(t+h\right)\right)} \end{array}$$

Also, we have

(47) $$V\left(t+h,X\left(t+h\right)\right)\leq \lambda_{2}{\left\|X\left(t+h\right)\right\|}^{2}$$

When V(X,t)=0, considering Equation (18) we obtain the following equation:

(48) X(t+h)=h[f(0)+Bg(t)]+o(h)

Consequently, we obtain the following equation:

(49) $${\left\|X\left(t+h\right)\right\|}^{2}=h^{2}\;{\left\|B\;g\left(t\right)\right\|}^{2}+h\;o\left(h\right)$$

Considering Equations (47) and (49), we have the following equation:

(50) $$\frac{1}{h^{2}}V\left(t+h,X\left(t+h\right)\right)\leq \lambda_{2}{\left\|g\left(t\right)\right\|}^{2}+\frac{o\left(h\right)}{h}$$

In other words

(51) $$\lim\;\underset{h\to 0^{+}}{\sup}\frac{1}{h}\sqrt{V\left(t+h,X\left(t+h\right)\right)}\leq \sqrt{\lambda_{2}}\;\left\|g\left(t\right)\right\|$$

Thus

(52) $$D^{+}W\leq \sqrt{\lambda_{2}}\left\|g\left(t\right)\right\|$$

Therefore, for any V(X(t),t), we get

(53) $$D^{+}W\leq -\varepsilon W+\sqrt{\lambda_{2}}\left\|g\left(t\right)\right\|$$

Using the comparison lemma, it follows that

(54) $$W\left(t\right)\leq e^{-t\varepsilon /2\lambda_{2}}W\left(0\right)+\sqrt{\frac{\lambda_{2}}{\lambda_{1}}}\;\int_{0}^{t}e^{-\left(t-\tau \right)\;\lambda_{3}/2\lambda_{2}}\left\|g\left(\tau \right)\right\|d\tau$$

Applying (25), we derive the following:

(55) $$\left\|X\left(t\right)\right\|\leq \sqrt{\frac{\lambda_{2}}{\lambda_{1}}}\left\|X\left(0\right)\right\|e^{-t\lambda_{3}/2\lambda_{2}}+\sqrt{\frac{\lambda_{2}}{\lambda_{1}}}\int_{0}^{t}e^{-\left(t-\tau \right)\;\lambda_{3}/2\lambda_{2}}\left\|g\left(\tau \right)\right\|d\tau$$

One can readily confirm that

(56) $$\left\|X_{0}\right\|\leq r\sqrt{\frac{\lambda_{1}}{\lambda_{2}}}\;\text{and}\;\underset{0\leq \sigma \leq t}{\sup}\left\|g\left(\sigma \right)\right\|\leq \frac{\lambda_{1}\lambda_{3}r}{\lambda_{2}\frac{1}{\sqrt{2}}}$$

Guarantee that ‖X(t)‖≤r, ensuring X(t) remains within the valid domain of the assumptions.

This outcome can once again be interpreted as a robustness characteristic of nominal systems with exponentially stable equilibria at the origin. It demonstrates that arbitrarily small, uniformly bounded perturbations do not cause significant steady‐state deviations from the origin. A high gain controller ensures the system's robustness. Table 1 presents the BS controller that we propose.

Remark 8 BIBO stability ensures that for any bounded input, the system produces a bounded output, making it a fundamental criterion for practical system performance. This type of stability guarantees predictable and controlled system behaviour, which is critical in real‐world applications where inputs are often constrained. BIBO stability is particularly useful for analysing and designing systems subject to external disturbances, ensuring that the system remains well‐behaved under such conditions. It provides a straightforward and intuitive stability measure, especially for systems in control engineering and signal processing, ensuring reliable and safe operation.

## 5 Application to Fractional Order Model

In this section, because of the advantages of fractional order systems, the application of the method to Kirschner's fractional‐order model is shown. It is proved that the controller ensures stability of the system in the presence of fractional model. Therefore, its fractional model is expressed in the following equation:

(57a) $$D^{q}x=cy-\mu_{2}x+\frac{p_{1}xz}{g_{1}+z}+u_{1},$$

(57b) $$D^{q}y=r_{2}\left(1-by\right)y-\frac{axy}{g_{2}+y},$$

(57c) $$D^{q}z=\frac{p_{2}xz}{g_{3}+y}-\mu_{3}z+u_{2},$$

where q is a fractional‐order operator set to 0.98 in simulations, $u_{1}$ is an external source of effector cells, $u_{2}$ represents an external input of effector cells.

Theorem 3 *The control input calculated by* Equations (13) and (15) *stabilise the tumour growth system modelled by fractional order such that the states* y,z *and* e *asymptotically converge to zero.*

Considering the definition of e by Equation (2), we can rewrite Eq. (57b) as follows:

(58) $$D^{q}y=r_{2}\left(1-by\right)y-\frac{1}{\alpha }\frac{ay}{g_{2}+y}\left(e+x^{*}\right).$$

Substituting $x^{*}$ by Equation (6) in Equation (58), we obtain the following equation:

(59) $$D^{q}y=-\frac{1}{\alpha }\frac{ay}{g_{2}+y}e-\frac{1}{\alpha }k_{1}\;y.$$

Fractional time derivative of definition (2), results

(60) $$D^{q}e=D^{q}x-D^{q}x^{*}.$$

Substituting Equation (57a) in the result, we obtain the following equation:

(61) $$D^{q}e=cy-\mu_{2}x+\frac{p_{1}xz}{g_{1}+z}+u_{1}-D^{q}x^{*}.$$

Fractional time derivative of $x^{*}$ by Equation (7) results

(62) $$D^{q}x^{*}=\left(2Ay+B\right)\;D^{q}y,$$

where A,B are calculated by Equation (8). Substituting Equation (59) in Equation (62), we obtain the following equation:

(63) $$D^{q}x^{*}=\left(2Ay+B\right)\;\left(-\frac{1}{\alpha }\frac{ay}{g_{2}+y}e-\frac{1}{\alpha }k_{1}y\right).$$

Substituting Equation (62) in Equation (61), we obtain the following equation:

(64) $$D^{q}e=cy-\mu_{2}x+\frac{p_{1}xz}{g_{1}+z}+u_{1}-\left(2Ay+B\right)\;\left(-\frac{ay}{g_{2}+y}e-k_{1}y\right).$$

Substituting the control input $u_{1}$ by Equation (13) in Equation (64), we obtain the following equation:

(65) $$D^{q}e=\frac{ay^{2}}{g_{2}+y}-k_{2}e.$$

Substituting the control input $u_{2}$ by Equation (15) in Equation (57c), we obtain the following equation:

(66) $$D^{q}z=-k_{3}z.$$

Choose the following Lyapunov function candidate:

(67) $$V=\frac{1}{2}e^{2}+\frac{1}{2}y^{2}+\frac{1}{2}z^{2}.$$

Fractional order derivative of Equation (67) results

(68) $$D^{q}V=e\;D^{q}e+y\;D^{q}y+z\;D^{q}z.$$

Substituting Equations (59), (65) and (66) in Equation (68) and simplifying the result, we obtain the following equation:

(69)

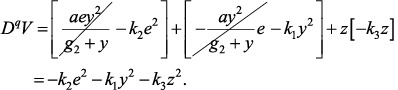




Equation (69) ensures $D^{q}V\leq 0$. Based on the above relation, the proposed control method ensures stability of the closed‐loop system. Moreover, the error of the outputs converges to zero asymptotically.

The proposed BS controller is summarised in Table 2.

**TABLE 2:** BS controller design.

Tumour growth model:	$\left\{\begin{array}{l} D^{q}x=cy-\mu_{2}x+\frac{p_{1}xz}{g_{1}+z}+u_{1}\\ D^{q}y=r_{2}\left(1-by\right)y-\frac{axy}{g_{2}+y}\\ D^{q}z=\frac{p_{2}xz}{g_{3}+y}-\mu_{3}z+u_{2} \end{array}\right.$ x: concentration of effector cells y: concentration of tumour cells z: concentration of single cancer site
Input controller:	$u_{1}=-cy+\mu_{2}x+\left(2Ay+B\right)\;\left(-\frac{ay}{g_{2}+y}e-k_{1}y\right)-\frac{p_{1}xz}{g_{1}+z}+\frac{ay}{g_{2}+y}y-k_{2}e$ $u_{2}=-\frac{p_{2}xz}{g_{3}+y}+\mu_{3}z-k_{3}z$ $k_{1},k_{2},k_{3}:$ control gains
Stability:	‐ Asymptotically stable

## 6 Simulation Results

In this section, the effectiveness of the presented controller is examined numerically. All simulations were performed in MATLAB software. Simulation results of similar research papers in the literature are presented for comparison. The design of a BS controller based on DRL is presented in Section 6.1. Furthermore, the exponential stability of our approach allows us to adjust the convergence rate (γ) of the controlled system. In Section 6.2, the results presented in this paper are juxtaposed with the results presented in ref. [35]. In Section 6.3, we compare the proposed results with the method presented in ref. [38] and the adaptive fuzzy controller presented in ref. [34]. Furthermore, we evaluate the robustness of the presented controller against uncertainties.

### 6.1 SAC‐Driven Parameter Tuning for BS Controller Development

The Markov Decision Process (MDP) serves as a widely used framework to structure reinforcement learning (RL) agents. It is characterised by a four‐element tuple, which defines its core components.

- **State** $s_{t}$ represents a collection of variables that define the system at time step t.
- **Action** $\delta_{t}$ represents the control signal applied at time step t.
- **Transition Probabilities** ρ indicate the likelihood of the environment moving from the current state $s_{t}$ to the next state $s_{t}^{\prime }$ when action $\delta_{t}$ is applied.
- **Reward** $r_{t}$ indicates how close the current state is to the target state at time step t.

In the reinforcement learning framework, the primary objective is to maximise the expected cumulative reward, $E\left[\sum_{k=0}^{T}\gamma^{k}r_{t+k}\right]$, accumulated from the environment over a time horizon T. The parameter γ∈[0,1] serves as the discount factor.

#### 6.1.1 Soft Actor‐Critic Approach

The Soft Actor‐Critic (SAC) algorithm, a leading method in deep reinforcement learning, effectively addresses high‐dimensional continuous action spaces through the use of soft policy iteration. Unlike traditional actor‐critic approaches, SAC maximises the entropy of the policy independently from the cumulative reward, allowing for more robust exploration and stability.

(70) $$Q_{\pi }\left(s_{t},\delta_{t}\right)=r_{t}+\gamma E_{s_{t+1},\delta_{t+1}}\left[Q\left(s_{t+1},\delta_{t+1}\right)-\alpha H\left(\pi_{\emptyset }\left(\cdot |s\right)\right)\right]$$

where H(·|s) represents entropy, $\pi_{\emptyset }$ is the policy following the distribution ∅, and α is an adjustable parameter.

**FIGURE 2:**
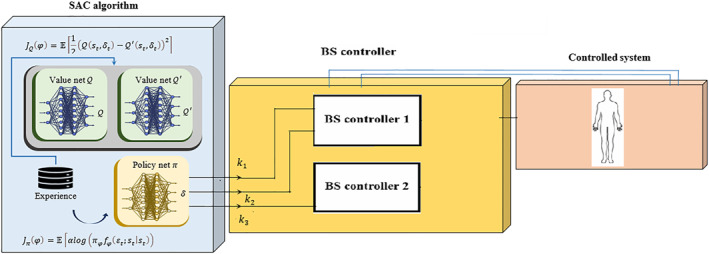
Structure of the BS controller developed using the SAC‐DRL algorithm for cancer immunotherapy.

The SAC architecture, shown in Figure 2, consists of three key components: a critic network that estimates the Q‐value, an actor (policy) network that generates action signals, and an experience buffer for storing tuples of state transitions ($D=\left[s_{t},\delta_{t},r_{t},s_{t+1}\right]$) used for training the networks. Additionally, a target Q network, which mirrors the structure of the Q network, is employed to enhance stability.

Using the tuples from the replay buffer D, the Q network can be updated with the following equation:

(71) $$J_{Q}\left(\varphi \right)=E_{\left(s_{t},\delta_{t}\right)∼D}\left[\frac{1}{2}{\left(Q\left(s_{t},\delta_{t}\right)-\left(r\left(s_{t},\delta_{t}\right)+\gamma E_{s_{t+1}∼p\left(s_{t},\delta_{t}\right)}\left[V_{\hat{\psi }}\left(s_{t+1}\right)\right]\right)\right)}^{2}\right]$$

where $V_{\hat{\psi }}\left(s_{t+1}\right)$ represents the target network.

For the policy function, the actor network's weights are adjusted using Kullback–Leibler (KL) divergence, expressed as follows:

(72) $$\pi =\arg\;\min_{\pi \in \Pi }D_{KL}\left(\pi_{\emptyset }\left(\cdot |s_{t}\right)\|\frac{\frac{1}{\alpha }\exp\left(Q_{s}\left(s_{t},\cdot \right)\right)}{Ζ\left(s_{t}\right)}\right)$$

where $D_{KL}\left(\cdot \right)$ represents the KL divergence and ${Ζ}^{\pi_{old}}\left(s_{t}\right)$ denotes the logarithm of the partition function, which serves to normalise the distribution. By minimising the Kullback–Leibler divergence and disregarding the term ${Ζ}^{\pi_{old}}\left(s_{t}\right)$ due to its minimal effect on the weight update for φ, the policy parameters are optimised as follows:

(73) $$J_{\pi }\left(\varphi \right)=E_{s_{t}∼D}\left[E_{s_{t}∼\pi_{\varphi }}\left[\alpha \;\log\left(\pi_{\varphi }\left(\delta_{t}|s_{t}\right)\right)-Q\left(s_{t},\pi_{\varphi }\left(s_{t}\right)\right)\right]\right]$$

Utilising a Gaussian distribution, the actions in SAC are generated through the following policy function:

(74) $$\delta_{t}=f_{\varphi }\left(\varepsilon_{t};s_{t}\right)$$

where $\varepsilon_{t}∼N\left(o,I\right)$.

Subsequently, the updated policy function is defined as follows:

(75) $$J_{\pi }\left(\varphi \right)=E_{s_{t}∼D,\varepsilon_{t}∼N\left(o,I\right)}\left[\alpha \;\log\left(\pi_{\varphi }f_{\varphi }\left(\varepsilon_{t};s_{t}|s_{t}\right)\right)-Q\left(s_{t},f_{\varphi }\left(\varepsilon_{t};s_{t}\right)\right)\right]$$

#### 6.1.2 SAC‐DRL Utilising a BS Controller

In this application, the controller gains are treated as the optimisation targets for the SAC algorithm. The three control coefficients that need to be refined are as follows:

(76) $$\left\{\begin{array}{l} \delta_{1}=k_{1}\\ \delta_{2}=k_{2}\\ \delta_{3}=k_{3} \end{array}\right.$$

Given that the primary control objective is to stabilise the system, the external source of effector cells ($u_{1}\left(t\right)$) and the influence of tumour infiltrating lymphocyte cells ($u_{2}\left(t\right)$), and the concentration of tumour cells y(t) are considered as part of the system state.

In the context of the optimisation problem, the reward function is formulated as follows:

(77) $$r_{t}=\left\{\begin{array}{cc} \frac{\sigma_{1}}{\left|u\left(t\right)-U_{Ref}\left(t\right)\right|} & if\;\left(u\left(t\right)-U_{Ref}\left(t\right)\right)<\varepsilon \\ -\sigma_{2}\left|u\left(t\right)-U_{Ref}\left(t\right)\right| & else \end{array}\right.$$

Here, $\sigma_{1}$ and $\sigma_{2}$ represent the weight coefficients. As specified, $\left|u\left(t\right)-U_{Ref}\left(t\right)\right|$ indicates that the SAC agent achieves u(t) a higher reward when the values of are closer to the nominal system input. Based on the term $-\sigma_{2}\left|u\left(t\right)-U_{Ref}\left(t\right)\right|$, the SAC agent incurs a significant penalty when u(t) deviates substantially from the nominal value, while only a minor penalty is applied for smaller deviations in u(t).

### 6.2 Comparison of the Results

**FIGURE 3:**
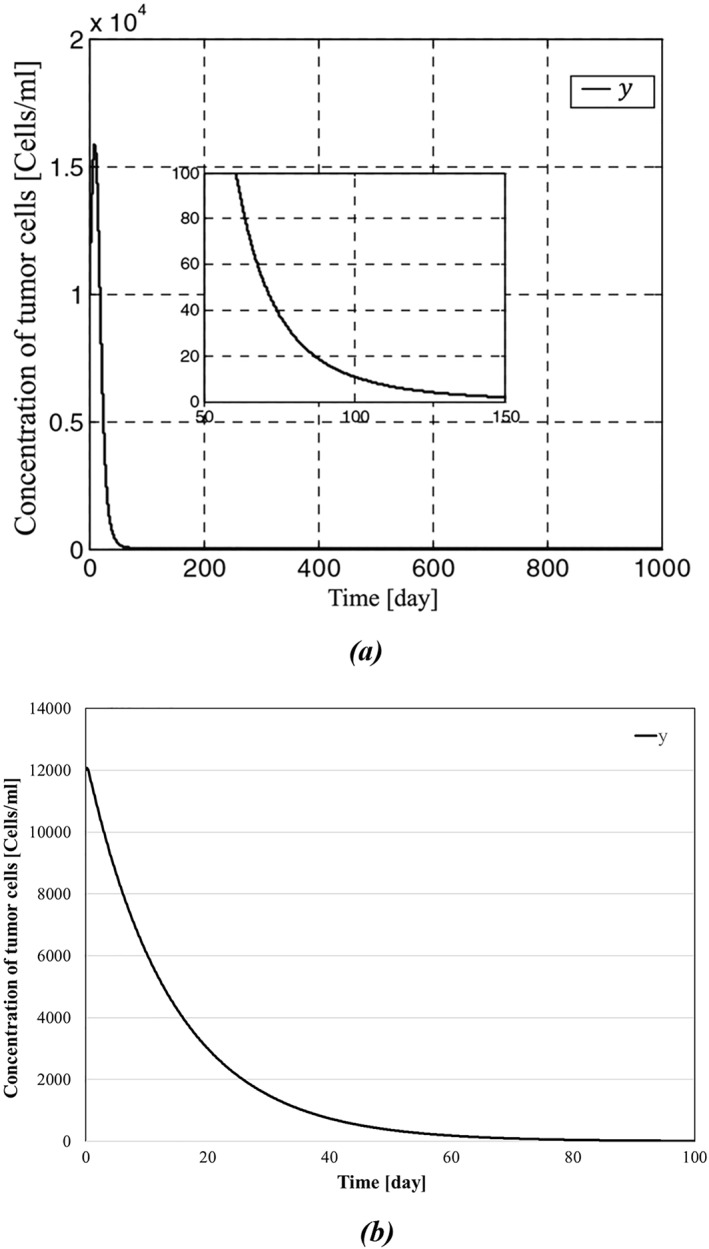
Concentration of tumour cells: (a) represents concentration of tumour cells presented in [35], and (b) depicts concentration of the tumour cells presented in this paper.

**FIGURE 4:**
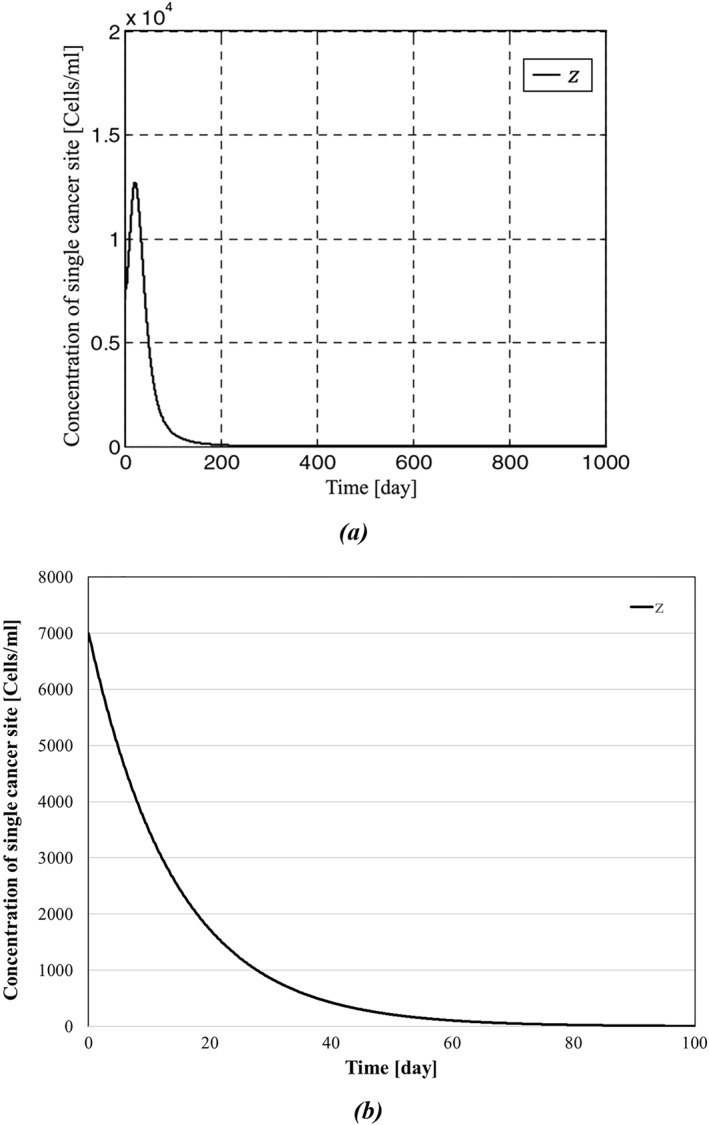
Concentration of single cancer site: (a) represents concentration of single cancer site presented in ref. [35], and (b) depicts concentration of single cancer site presented in this paper.

**FIGURE 5:**
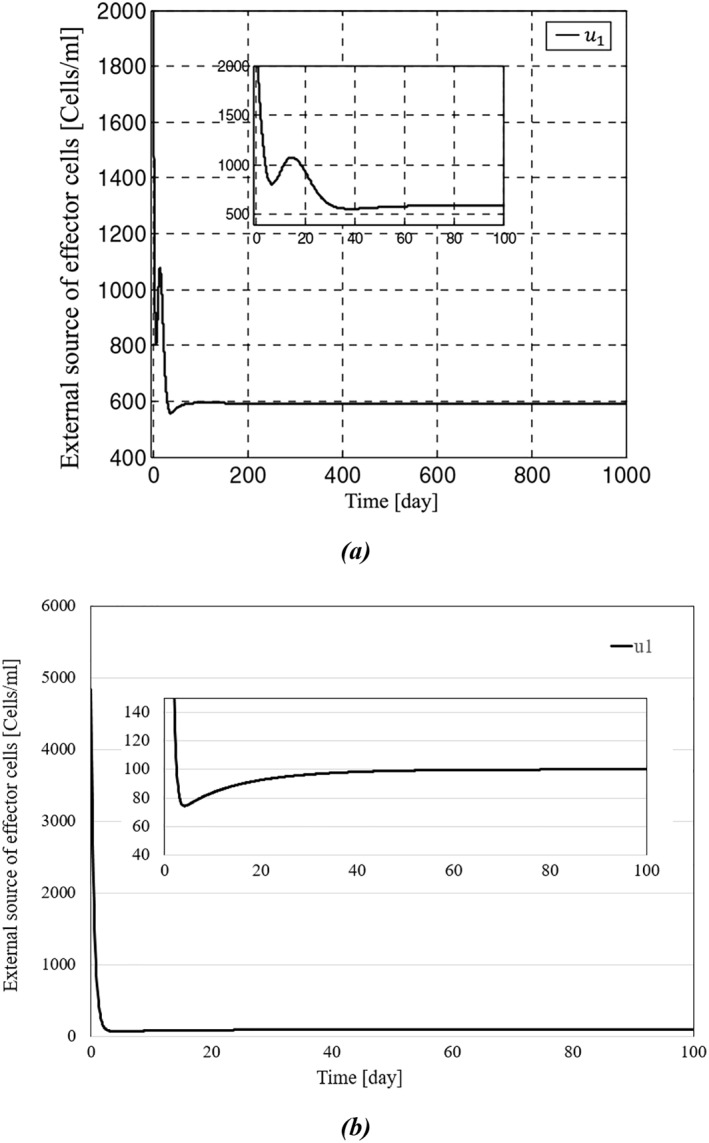
Time history of external source of effector cells: (a) represents external source of effector cells presented in ref. [35], and (b) shows external source of effector cells presented in this paper.

**FIGURE 6:**
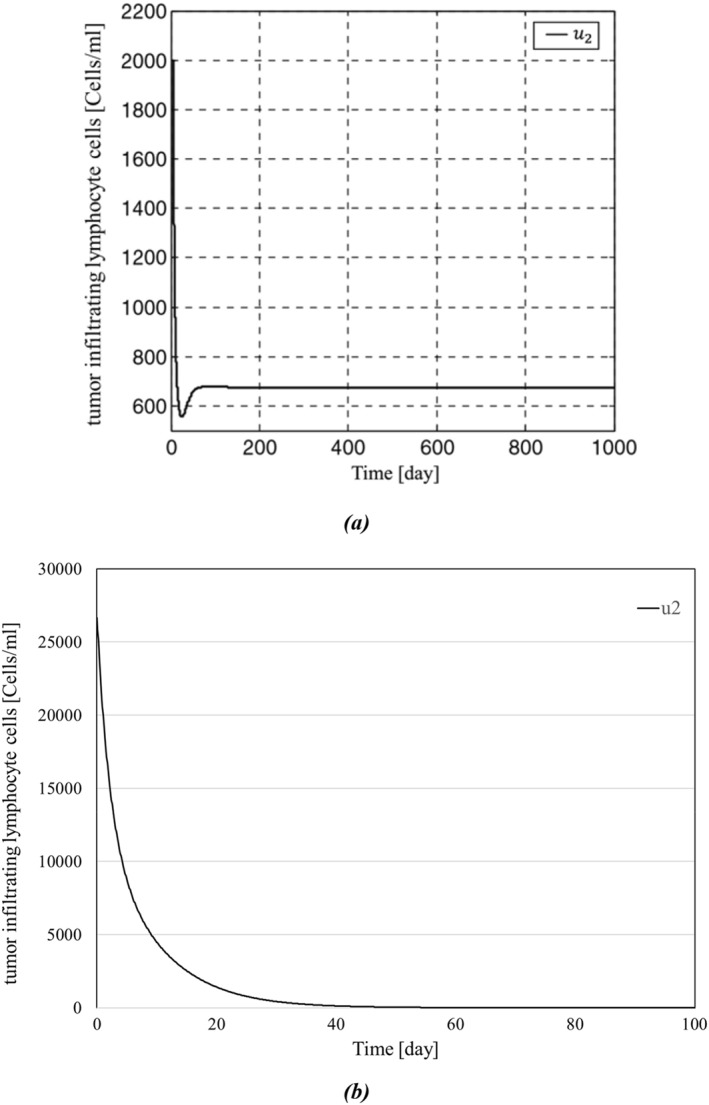
Time history of tumour infiltrating lymphocyte cells: (a) tumour infiltrating lymphocyte cells presented in ref. [35], and (b) tumour infiltrating lymphocyte cells presented in this paper.

The results of simulations between the proposed backstepping controller and the controller proposed in [35] are shown in Figures 3, 4, 5, 6. As it can be seen in Figure 3a, the concentration of the tumour cells in [35] has reached zero after a span of roughly 150 days; in contrast, Figure 3b shows a more effective eradication of the tumour cells after a span of 50 days. In addition to earlier elimination of the tumour cells, it can be observed in Figure 4b that IL‐2 has reached zero after a span of 40 days, which is more efficient compared to Figure 4a. The initial conditions for the system states are set as

(78) x(0)=10000,y(0)=12000,z(0)=7000

Consequently, the initial magnitude of $u_{1},u_{2}$ is determined based on the control inputs. Since the control methods employed in the proposed approach and [35] differ, the initial magnitudes of $u_{1}$ and $u_{2}$ will also differ.

**FIGURE 7:**
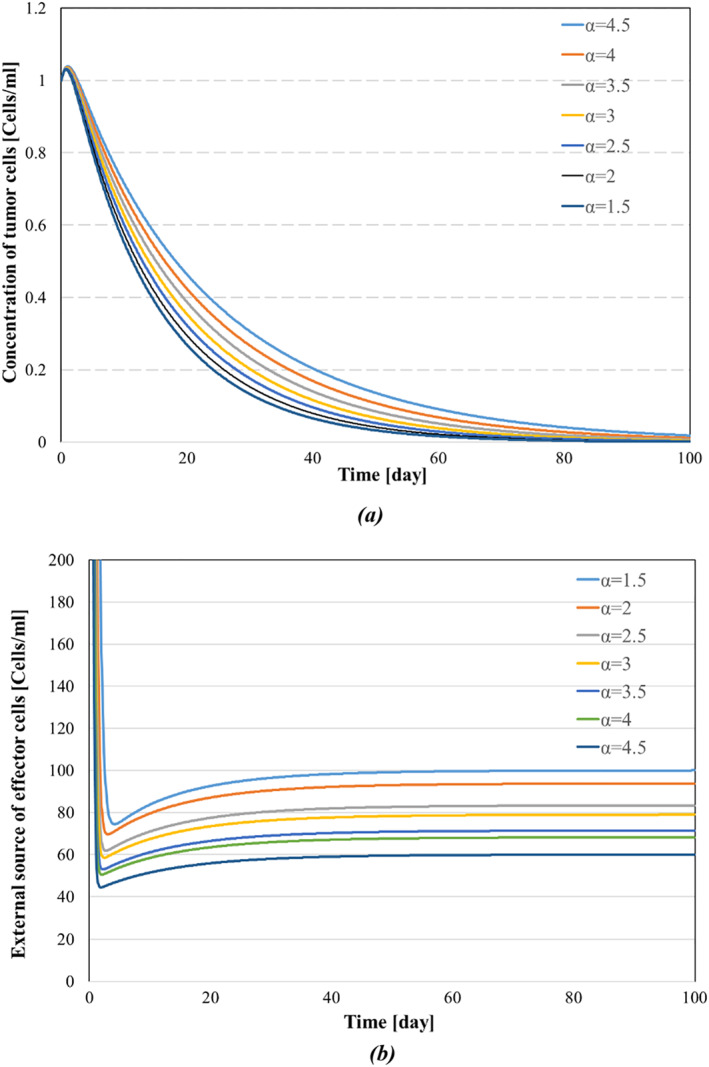
Effect of parameter α on control performance: (a) tumour cell concentration, and (b) external source of effector cells.

The backstepping controller curves of $u_{1}$ and $u_{2}$ are shown in Figures 5 and 6, respectively. In Figure 5a, the external source of effector cells settles at around 80 days, similar to our controller's response shown in Figure 5b. However, Figure 5a exhibits more oscillations in the transient response, while our model has a smoother response with fewer oscillations. Moreover, Figure 6b demonstrates that $u_{2}$, after a short amount of time, has reached zero in a way that will efficiently lower the cost, whereas Figure 6a shows the inability of $u_{2}$ to reach zero. As mentioned in Remark 6, as the states y and z, along with the costate e, converge to zero, the control input $u_{2}$ tends to zero, and the steady‐state magnitude of $u_{1}$ can be sufficiently reduced by properly tuning the control gains. The continuous and permanent application of an external source of effector cells to the system, without converging to zero, poses significant risks to patients and is not practical, as indicated by [46]. In this paper, we demonstrate that by appropriately assigning the gain α, we can reduce the magnitude of the external source of effector cells. For instance, in Figure 7a,b, we numerically analyse the effects of parameter α on the convergence rate of tumour cell concentration and the steady‐state magnitude of the external source of effector cells, as discussed in Remark 6.

### 6.3 Evaluation of Comparative Results

**FIGURE 8:**
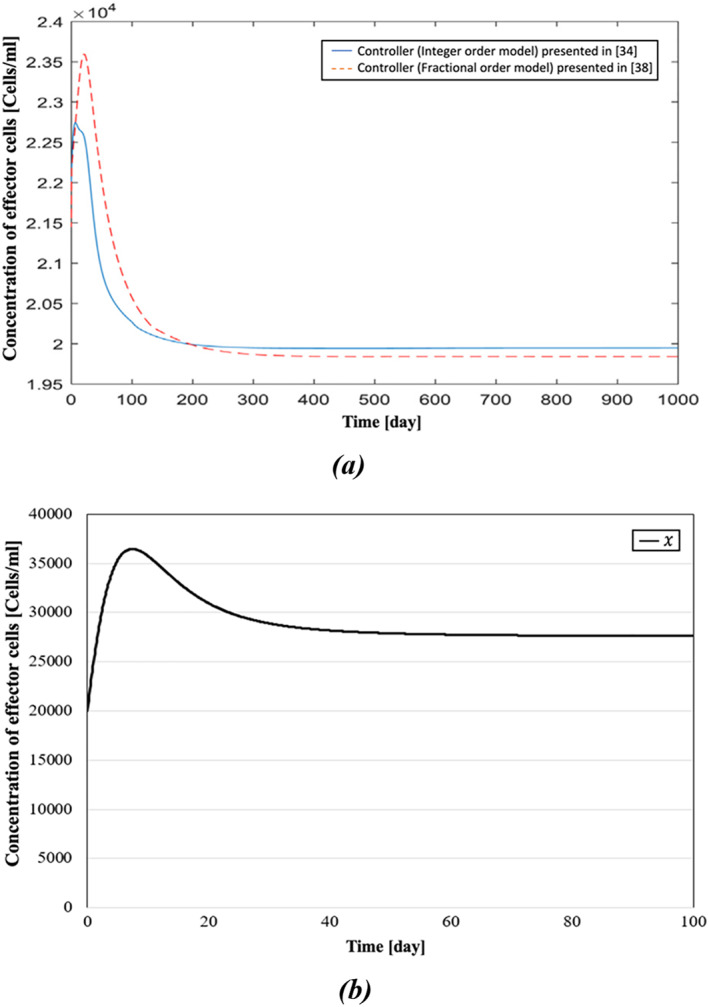
Concentration of the effector cells: (a) comparative demonstration of the concentration of the effector cells presented in ref. [38] and presented in ref. [34], and (b) concentration of the effector cells presented in this paper.

**FIGURE 9:**
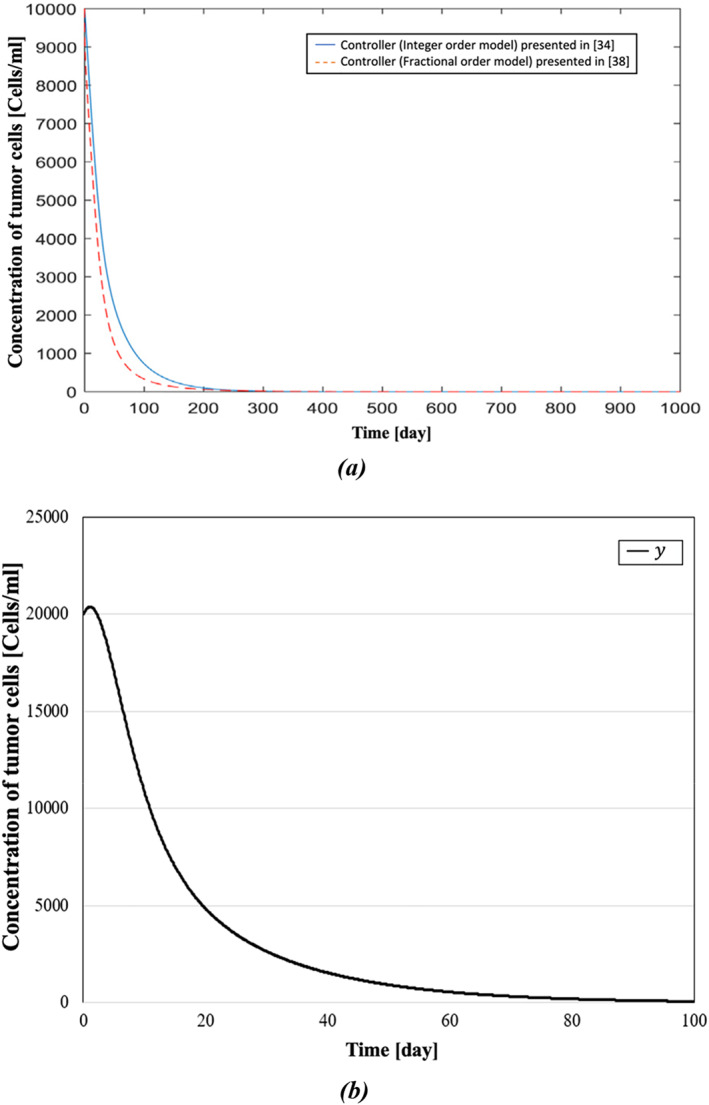
Concentration of the tumour cells: (a) comparative demonstration of the concentration of the tumour cells presented in ref. [38] and presented in ref. [34], and (b) concentration of the tumour cells presented in this paper.

**FIGURE 10:**
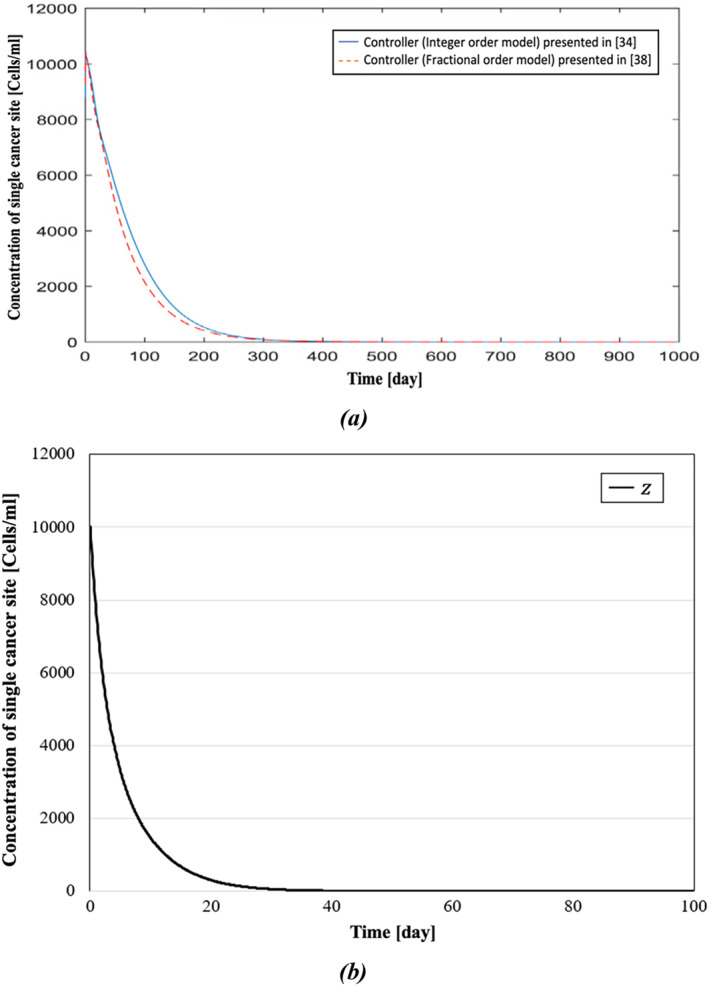
Concentration of single cancer site: (a) comparative demonstration of the concentration of single cancer site presented in ref. [38] and presented in ref. [34] and (b) concentration of single cancer site presented in this paper.

In ref. [38], Sarhaddi and Yaghoobi compared their method with the integer‐order model presented in [34]. It has been shown that the fractional model could be a better mathematical model for cancer dynamics compared to the integer‐order model because fractional derivatives have hereditary and memory properties of many physical phenomena. In this paper, for modelling the reciprocal effects of the tumour and immune system, the fractional‐order differential equations have also been used because of their aforementioned memory properties. A comparative display of the simulation results is presented in Figures 8, 9, 10. In Figure 8a, the concentration of effector cells in both models takes up to around 280–300 days to settle. In contrast, the concentration of effector cells in our model, shown in Figure 8b, reaches a fixed value after roughly 70 days. In Figure 9b, it can be observed that, using the presented controller, the tumour cells have reached zero after a span of 80 days, whereas Figure 9a shows that it takes nearly four times longer for tumour cells to reach zero compared to Figure 9b. Moreover, while it takes more than 350 days for IL‐2 cells of both models in Figure 10a to reach zero, Figure 10b shows that it has reached zero only after 40 days.

In Table 3, results from refs. [34, 35, 38] and this paper are compared. As illustrated in the dynamic responses of Figures 8, 9, 10, the presented BS‐based controller demonstrates superior control performance, achieving quicker settling times for the dynamic responses. Although theoretically and numerically our immunotherapy protocol demonstrates strong performance, with the controlled system being exponentially stable and robust against uncertainties, clinical trials using this protocol could be conducted to fully understand its efficacy in real‐world scenarios.

**TABLE 3:** Comparison of the performance of controlled systems.

	Proposed controller	Ref. [38]	Ref. [34]	Ref. [35]
Stability results	Exponential	Asymptotic	Asymptotic	Asymptotic
Robustness to uncertainties	✓	×	×	×
Requiring knowledge of disturbance	×	✓	✓	✓
Time for tumour cells to converge to zero (days)	50	280	300	150
Remaining effector cells after therapy (cells/mL)	27,000	19,900	20,150	N/A

### 6.4 Stability and Robustness Analysis Considering Uncertainties, Measurement Delays, and Discretisation

**FIGURE 11:**
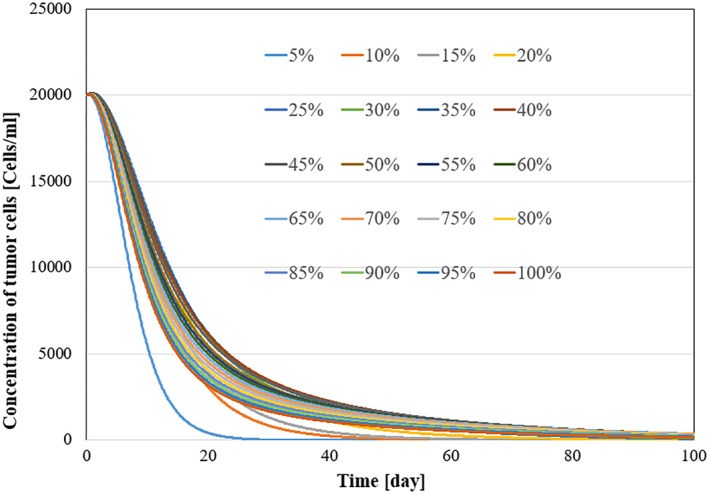
Concentration of tumour cells with 10%–100% system parameter uncertainties.

**FIGURE 12:**
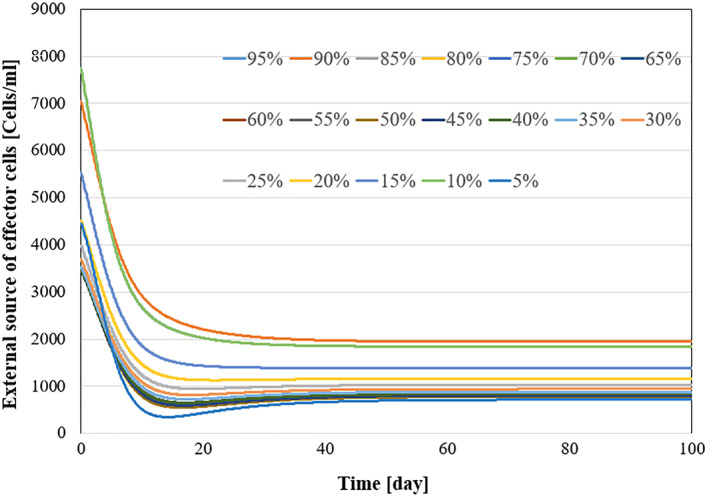
Time history of external source of effector cells with 10%–100% system parameter uncertainties.

**FIGURE 13:**
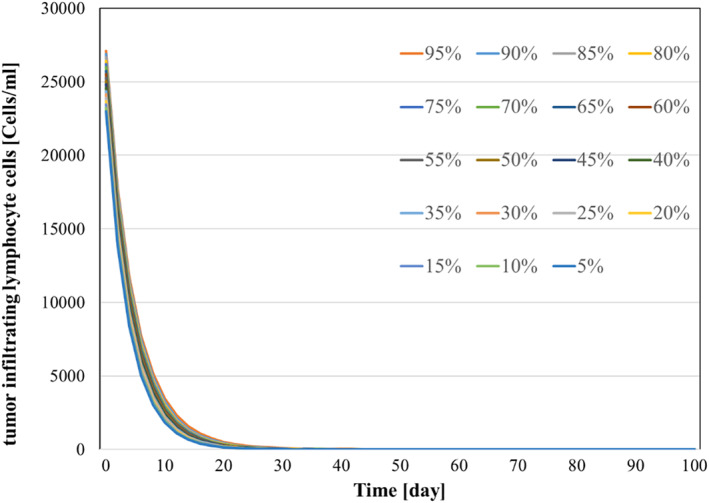
Time history of external source of effector cells with 10%–100% system parameter uncertainties.

**FIGURE 14:**
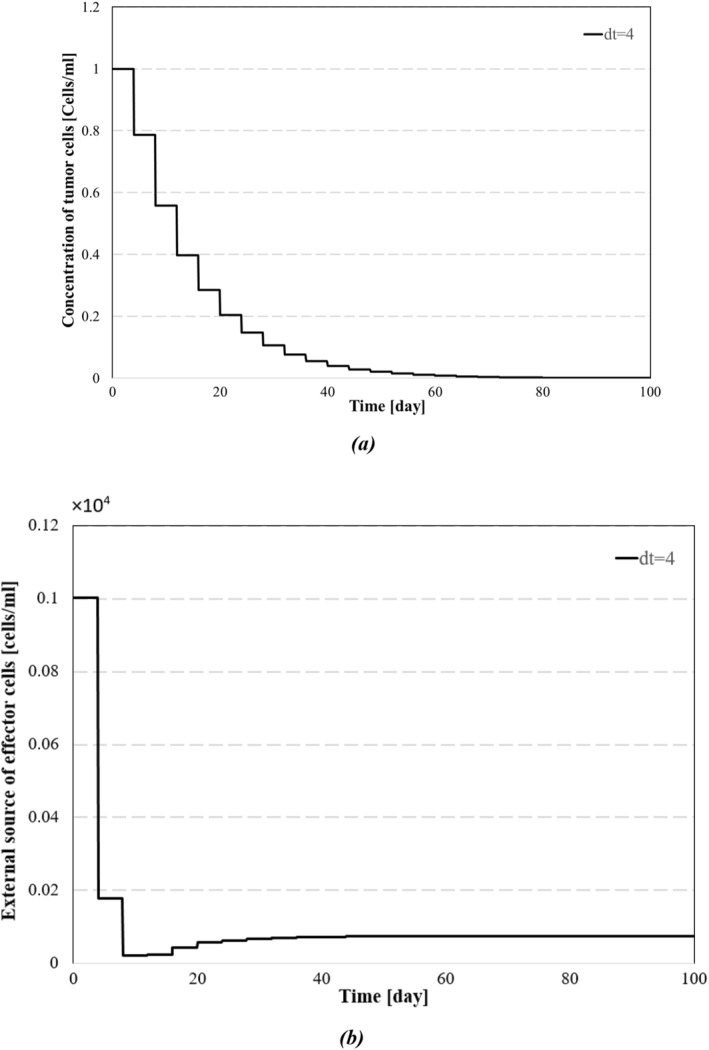
Effect of discretisation in the control performance: (a) a comparative demonstration of the concentration of the effector cells and (b) external source of effector cells.

All system parameters used in the simulation are listed in Table 4. Kirschner et al. [9] derived these parameters by drawing data from medical literature and performing sensitivity analysis. For additional details on parameter derivation, please refer to [9]. To demonstrate the robustness of the system against parameter uncertainties, we simulate the proposed controller against 10%–100% uncertainties in the system parameters. The time history of the resulting tumour volume, inhibitor level, and injection rate are shown individually in Figures 11, 12, 13, 14. It can be seen that the controller is robust against uncertainties in system parameters. In addition, it can be observed that a higher control injection rate is needed to control the system against more uncertainties in the system parameters.

**TABLE 4:** System parameters.

Parameter		Symbols	Values
Antigenicity		c	0≤c≤0.05
Death rate of immune cells		$\mu_{2}$	0.03
The proliferation rate of immune cells		$p_{1}$	0.1245
Half‐sat. for cancer clearance		$g_{2}$	$1\times 10^{5}$
Cancer growth rate		$r_{2}$	0.18
Logistic growth of cancer capacity		b	$1\times 10^{-9}$
The half‐life of an effector molecule		$\mu_{3}$	10
The production rate of an effector molecule		$p_{2}$	5
Half sat. for proliferation term		$g_{1}$	$2\times 10^{7}$
Cancer clearance term		a	1
Half sat. of production		$g_{3}$	$1\times 10^{3}$

In practice, the controller operates based on discrete measurements of system output as given by the following equation:

(79) $$\begin{array}{c} u_{1i}=-cy_{i}+\mu_{2}x_{i}+\frac{1}{\alpha }\left(2Ay_{i}+B\right)\;\left(-\frac{1}{\alpha }\frac{ay_{i}}{g_{2}+y_{i}}e_{i}-\frac{1}{\alpha }k_{1}y_{i}\right)-\frac{p_{1}x_{i}z_{i}}{g_{1}+z_{i}}+\frac{ay_{i}}{g_{2}+y_{i}}y_{i}-k_{2}e_{i}.\\ u_{2i}=-\frac{p_{2}x_{i}z_{i}}{g_{3}+y_{i}}+\mu_{3}z_{i}-k_{3}z_{i}. \end{array}$$

where $x_{i}=x\left(t_{i}\right),y_{i}=y\left(t_{i}\right),z_{i}=z\left(t_{i}\right),$ represent the discrete state observations and $u_{1i}=u_{1}\left(t_{i}\right),u_{2i}=u_{2}\left(t_{i}\right),$ represent the discrete inputs. The time instances are given by $t_{i}=t_{0}+i\;\Delta t$, where $t_{0}$ is the initial time and Δt represents the time increment. The designed control inputs incorporate the system state variables, meaning that these variables must be continuously measured and fed back to the controller. To assess the robustness of the proposed method under discrete state measurements, we analyse its effectiveness in Figure 14a,b, considering the impact of discrete system state observations.

Remark 9 In cancer control, activated immune cell concentrations are measured via blood tests, flow cytometry, immunohistochemistry (IHC), biopsy analysis, molecular/genetic techniques, and imaging. Tumour cell quantification, essential for diagnosis, treatment monitoring, and disease progression, employs biopsy/histopathology, flow cytometry, imaging, and molecular methods. Interleukin‐2 (IL‐2), a key cytokine for immune regulation and T cell activation, is assessed using ELISA, multiplex immunoassays, and flow cytometry to evaluate immune responses, immunotherapy efficacy, and inflammation. These methods collectively provide insights into immune activity, tumour burden, and cytokine dynamics in cancer research and management.

## 7 Conclusions

In this paper, a novel robust controller has been formulated for stabilising MIMO cancer immunotherapy with application to a tumour‐immunity fractional order model. Unlike previous studies, the controlled system is proved to be exponentially stable using the Lyapunov theorem. Because of the exponential stability, the convergence rate of the tumour volume can be controlled by adjusting the control gains. Furthermore, we mathematically demonstrate bounded input‐bounded output (BIBO) stability using the Lyapunov theorem and confirm the system's robustness against uncertainties through simulations with system parametric uncertainties ranging from 10% to 100%, proving that the system remains robust and stable. The simulation results show the effectiveness and superiority of the proposed controller compared to previous related studies, with tumour cells entirely eradicated within 50 days. Future studies could explore the efficacy of the proposed controller across other cancer therapy modalities.

## Author Contributions

**Mohamadreza Homayounzade:** conceptualization, formal analysis, investigation, methodology, project administration, resources, software, validation, supervision, visualization, writing – original draft, writing – review and editing. **Shayan Sajadian:** formal analysis, investigation, methodology, software, validation, data curation, visualization, writing – original draft, writing – review and editing.

## Conflicts of Interest

The authors declare no conflicts of interest.

## Data Availability

The data that support the findings of this study are available from the corresponding author upon reasonable request.

## References

[1] H. Sung , J. Ferlay , R. L. Siegel , et al., “Global Cancer Statistics 2020: GLOBOCAN Estimates of Incidence and Mortality Worldwide for 36 Cancers in 185 Countries,” CA: A Cancer Journal for Clinicians 71, no. 3 (2021): 209–249, 10.3322/caac.21660. 33538338

[2] L. G. De Pillis and A. Radunskaya , “A Mathematical Tumor Model With Immune Resistance and Drug Therapy: An Optimal Control Approach,” Computational and Mathematical Methods in Medicine 3, no. 2 (2001): 79–100, 10.1080/10273660108833067.

[3] M. Mamat , A. K. Subiyanto , and A. Kartono , “Mathematical Model of Cancer Treatments Using Immunotherapy, Chemotherapy and Biochemotherapy,” Applied Mathematical Sciences 7 (2013): 247–261, 10.12988/ams.2013.13023.

[4] O. G. Isaeva and V. A. Osipov , “Different Strategies for Cancer Treatment: Mathematical Modelling,” Computational and Mathematical Methods in Medicine 10, no. 4 (2009): 253–272, 10.1080/17486700802536054.

[5] F. A. Rihan and G. Velmurugan , “Dynamics of Fractional‐Order Delay Differential Model for Tumor‐Immune System,” Chaos, Solitons and Fractals 132 (2020): 109592, 10.1016/j.chaos.2019.109592.

[6] H. Hassani , Z. Avazzadeh , P. Agarwal , et al., “A Study on Fractional Tumor‐Immune Interaction Model Related to Lung Cancer via Generalized Laguerre Polynomials,” BMC Medical Research Methodology 23, no. 1 (2023): 189, 10.1186/s12874-023-02006-3. 37605131 PMC10440950

[7] S. Bunimovich‐Mendrazitsky , H. Byrne , and L. Stone , “Mathematical Model of Pulsed Immunotherapy for Superficial Bladder Cancer,” Bulletin of Mathematical Biology 70, no. 7 (2008): 2055–2076, 10.1007/s11538-008-9344-z. 18716846

[8] J. Yang , S. Tang , and R. A. Cheke , “Modelling Pulsed Immunotherapy of Tumour–Immune Interaction,” Mathematics and Computers in Simulation 109 (2015): 92–112, 10.1016/j.matcom.2014.09.001.

[9] D. Kirschner and J. C. Panetta , “Modeling Immunotherapy of the Tumor–Immune Interaction,” Journal of Mathematical Biology 37, no. 3 (1998): 235–252, 10.1007/s002850050127. 9785481

[10] F. A. Rihan and N. F. Rihan , “Dynamics of Cancer‐Immune System With External Treatment and Optimal Control,” Journal of Cancer Science & Therapy 8, no. 10 (2016): 257–261, 10.4172/1948-5956.1000423.

[11] X. Wu , Q. Liu , K. Zhang , M. Cheng , and X. Xin , “Optimal Switching Control for Drug Therapy Process in Cancer Chemotherapy,” European Journal of Control 42 (2018): 49–58, 10.1016/j.ejcon.2018.02.004.

[12] F. F. Teles and J. M. Lemos , “Cancer Therapy Optimization Based on Multiple Model Adaptive Control,” Biomedical Signal Processing and Control 48 (2019): 255–264, 10.1016/j.bspc.2018.09.016.

[13] R. F. Bandpey and A. A. Kalat , “An Observer‐Based Adaptive Fuzzy Control for Prescribing Drug Dosage in Cancer Treatment,” Biocybernetics and Biomedical Engineering 42, no. 4 (2022): 1137–1148, 10.1016/j.bbe.2022.09.004.

[14] F. Subhan , M. A. Aziz , I. U. Khan , et al., “Cancerous Tumor Controlled Treatment Using Search Heuristic (GA)‐based Sliding Mode and Synergetic Controller,” Cancers 14, no. 17 (2022): 4191, 10.3390/cancers14174191. 36077727 PMC9454425

[15] M. Nazari , A. Ghaffari , and F. Arab , “Finite Duration Treatment of Cancer by Using Vaccine Therapy and Optimal Chemotherapy: State‐dependent Riccati Equation Control and Extended Kalman Filter,” Journal of Biological Systems 23, no. 1 (2015): 1–29, 10.1142/S0218339015500011.

[16] M. Nazari , N. Babaei , and M. Nazari , “Nonlinear SDRE Based Adaptive Fuzzy Control Approach for Age‐specific Drug Delivery in Mixed Chemotherapy and Immunotherapy,” Biomedical Signal Processing and Control 68 (2021): 102687, 10.1016/j.bspc.2021.102687.

[17] N. H. Sweilam , S. M. Al‐Mekhlafi , A. O. Albalawi , and J. T. Machado , “Optimal Control of Variable‐Order Fractional Model for Delay Cancer Treatments,” Applied Mathematical Modelling 89 (2021): 1557–1574, 10.1016/j.apm.2020.08.012.

[18] F. S. Lobato , V. S. Machado , and Jr V. Steffen , “Determination of an Optimal Control Strategy for Drug Administration in Tumor Treatment Using Multi‐Objective Optimization Differential Evolution,” Computer Methods and Programs in Biomedicine 131 (2016): 51–61, 10.1016/j.cmpb.2016.04.004. 27265048

[19] A. Cappuccio , F. Castiglione , and B. Piccoli , “Determination of the Optimal Therapeutic Protocols in Cancer Immunotherapy,” Mathematical Biosciences 209, no. 1 (2007): 1–3, 10.1016/j.mbs.2007.02.009. 17416392

[20] H. Hassani , J. T. Machado , and S. Mehrabi , “An Optimization Technique for Solving a Class of Nonlinear Fractional Optimal Control Problems: Application in Cancer Treatment,” Applied Mathematical Modelling 93 (2021): 868–884, 10.1016/j.apm.2021.01.004.

[21] P. Khalili , S. Zolatash , R. Vatankhah , and S. Taghvaei , “Optimal Control Methods for Drug Delivery in Cancerous Tumour by Anti‐angiogenic Therapy and Chemotherapy,” IET Systems Biology 15, no. 1 (2021): 14–25, 10.1049/syb2.12010. 33491873 PMC8675840

[22] X. Ying and B. Li , “Machine‐learning Modeling for Personalized Immunotherapy‐An Evaluation Module,” Biomedical Journal of Scientific and Technical Research 47, no. 2 (2022): 38211, 10.26717/BJSTR.2022.47.007462. 37817882

[23] C. Arunkumar and S. Ramakrishnan , “Prediction of Cancer Using Customised Fuzzy Rough Machine Learning Approaches,” Healthcare Technology Letters 6, no. 1 (2019): 13–18, 10.1049/htl.2018.5055. 30881694 PMC6407447

[24] N. Jafarpisheh and M. Teshnehlab , “Cancers Classification Based on Deep Neural Networks and Emotional Learning Approach,” IET Systems Biology 12, no. 6 (2018): 258–263, 10.1049/iet-syb.2018.5002. 30472689 PMC8687421

[25] A. Dhillon and A. Singh , “eBreCaP: Extreme Learning‐based Model for Breast Cancer Survival Prediction,” IET Systems Biology 14, no. 3 (2020): 160–169, 10.1049/iet-syb.2019.0087. 32406380 PMC8687246

[26] H. Naderi , M. Mehrabi , and M. T. Ahmadian , “Adaptive Fuzzy Controller Design of Drug Dosage Using Optimal Trajectories in a Chemoimmunotherapy Cancer Treatment Model,” Informatics in Medicine Unlocked 27 (2021): 100782, 10.1016/j.imu.2021.100782.

[27] F. Zouari , “Neural Network Based Adaptive Backstepping Dynamic Surface Control of Drug Dosage Regimens in Cancer Treatment,” Neurocomputing 366 (2019): 248–263, 10.1016/j.neucom.2019.07.096.

[28] E. Ahmadi , J. Zarei , R. Razavi‐Far , and M. Saif , “A Dual Approach for Positive T–S Fuzzy Controller Design and its Application to Cancer Treatment under Immunotherapy and Chemotherapy,” Biomedical Signal Processing and Control 58 (2020): 101822, 10.1016/j.bspc.2019.101822.

[29] M. E. Karar , A. H. El‐Garawany , and M. El‐Brawany , “Optimal Adaptive Intuitionistic Fuzzy Logic Control of Anti‐cancer Drug Delivery Systems,” Biomedical Signal Processing and Control 58 (2020): 101861, 10.1016/j.bspc.2020.101861.

[30] M. Sharifi and H. Moradi , “Nonlinear Composite Adaptive Control of Cancer Chemotherapy With Online Identification of Uncertain Parameters,” Biomedical Signal Processing and Control 49 (2019): 360–374, 10.1016/j.bspc.2018.07.009.

[31] A. P. Shahri , S. Haghighatnia , R. K. Moghaddam , and H. R. Kobravi , “Control the Tumour Growth via Sliding Mode Control,” International Journal of Medical Engineering and Informatics 9, no. 2 (2017): 101–109, 10.1504/IJMEI.2017.083093.

[32] P. Khalili , R. Vatankhah , and S. Taghvaei , “Optimal Sliding Mode Control of Drug Delivery in Cancerous Tumour Chemotherapy Considering the Obesity Effects,” IET Systems Biology 12, no. 4 (2018): 185–189, 10.1049/iet-syb.2017.0094. 33451184 PMC8687235

[33] E. S. Ghasemabad , I. Zamani , H. Tourajizadeh , M. Mirhadi , and Z. G. Zarandi , “Design and Implementation of an Adaptive Fuzzy Sliding Mode Controller for Drug Delivery in Treatment of Vascular Cancer Tumours and its Optimisation Using Genetic Algorithm Tool,” IET Systems Biology 16, no. 6 (2022): 201–219, 10.1049/syb2.12051. 36181296 PMC9675414

[34] H. Nasiri and A. A. Kalat , “Adaptive Fuzzy Back‐Stepping Control of Drug Dosage Regimen in Cancer Treatment,” Biomedical Signal Processing and Control 42 (2018): 267–276, 10.1016/j.bspc.2018.02.001.

[35] S. Khorashadizadeh and A. Akbarzadeh Kalat , “Adaptive Back‐stepping Cancer Control Using Legendre Polynomials,” IET Systems Biology 14, no. 1 (2020): 8–15, 10.1049/iet-syb.2019.0038. 31931476 PMC8687416

[36] M. M. Zirkohi and T. Kumbasar , “Adaptive Backstepping Controller Design for MIMO Cancer Immunotherapy Using Laguerre Polynomials,” Journal of the Franklin Institute 357, no. 8 (2020): 4664–4679, 10.1016/j.jfranklin.2020.02.007.

[37] M. Nazari , M. Nazari , and M. H. Noori Skandari , “Pseudo‐spectral Method for Controlling the Drug Dosage in Cancer,” IET Systems Biology 14, no. 5 (2020): 261–270, 10.1049/iet-syb.2020.0054. 33095747 PMC8687205

[38] M. Sarhaddi and M. Yaghoobi , “A New Approach in Cancer Treatment Regimen Using Adaptive Fuzzy Back‐Stepping Sliding Mode Control and Tumor‐Immunity Fractional Order Model,” Biocybernetics and Biomedical Engineering 40, no. 4 (2020): 1654–1665, 10.1016/j.bbe.2020.09.003.

[39] O. Yousefi , P. Azami , M. Sabahi , R. Dabecco , B. Adada , and H. Borghei‐Razavi , “Management of Optic Pathway Glioma: A Systematic Review and Meta‐Analysis,” Cancers 14, no. 19 (2022): 4781, 10.3390/cancers14194781. 36230704 PMC9563939

[40] Z. Avazzadeh , H. Hassani , M. J. Ebadi , et al., “Generalized Lerch Polynomials: Application in Fractional Model of CAR‐T Cells for T‐Cell Leukemia,” European Physical Journal Plus 138, no. 12 (2023): 1152, 10.1140/epjp/s13360-023-04786-5.

[41] M. Homayounzade , M. Homayounzadeh , and M. H. Khooban , “Robust Positive Control of Tumour Growth Using Angiogenic Inhibition,” IET Systems Biology 17, no. 5 (2023): 288–301, 10.1049/syb2.12076. 37787083 PMC10580019

[42] A. J. Abougarair , M. Bakouri , A. Alduraywish , et al., “Optimizing Cancer Treatment Using Optimal Control Theory,” AIMS Mathematics 9, no. 11 (2024): 31740–31769, 10.3934/math.20241526.

[43] C. K. Nanditha and M. P. Rajan , “An Adaptive Pharmacokinetic Optimal Control Approach in Chemotherapy for Heterogeneous Tumor,” Journal of Biological Systems 30, no. 3 (2022): 529–551, 10.1142/s0218339022500188.

[44] M. O. Olayiwola and A. I. Alaje , “A Space‐Time Caputo Fractional Order and Modified Homotopy Perturbation Method for Evaluating the Pathological Response of Tumor‐Immune Cells,” Healthcare Analytics 5 (2024): 100325, 10.1016/j.health.2024.100325.

[45] M. Homayounzade , “Predictor‐Based Output Feedback Control of Tumour Growth With Positive Input: Application to Antiangiogenic Therapy,” IET Systems Biology 19, no. 1 (2025), 10.1049/syb2.70005. PMC12019309 40270482

[46] S. L. Maude , T. W. Laetsch , J. Buechner , et al., “Tisagenlecleucel in Children and Young Adults with B‐Cell Lymphoblastic Leukemia,” New England Journal of Medicine 378, no. 5 (2018): 439–448, 10.1056/nejmoa1709866. 29385370 PMC5996391

